# Knowledge, socio-cognitive perceptions and the practice of hand hygiene and social distancing during the COVID-19 pandemic: a cross-sectional study of UK university students

**DOI:** 10.1186/s12889-021-10461-0

**Published:** 2021-03-01

**Authors:** Christine Barrett, Kei Long Cheung

**Affiliations:** Department of Health Sciences, Brunel University London, Uxbridge, UB8 3PH UK

**Keywords:** COVID-19 pandemic, Health behaviours, Social distancing, Hand hygiene, University students, Determinants, I-change

## Abstract

**Background:**

During the first wave of the COVID-19 pandemic, social distancing and hand hygiene have been the primary means of reducing transmission in the absence of effective treatments or vaccines, but understanding of their determinants is limited. This study aimed to investigate knowledge and socio-cognitive perceptions, and their associations with such protective behaviours, in UK university students.

**Methods:**

A cross-sectional online survey of 293 students was undertaken on 13 May 2020. Survey questions addressed demographics, knowledge of the disease and effectiveness of the protective measures, risk perception, socio-cognitive perceptions (e.g. attitude, social support, and self-efficacy), habit, time factors and trust, as well as the hand hygiene and social distancing behaviours. Multiple linear regression was used to identify the strongest associations of potential determinants with behaviour.

**Results:**

Participants reported high levels of social distancing with 88.9% answering “Mostly” or “Always” for every activity, but only 42.0% reporting the same for all hand hygiene activities. Knowledge of the effectiveness of each activity in preventing transmission was high, with 90.7% and 93.5% respectively identifying at least 7 of 8 hand hygiene or 9 of 10 social distancing activities correctly. Habit (*β* = 0.39, *p* = 0.001) and time factors (*β* = 0.28, *p* = 0.001) were the greatest contributors to unique variance in hand hygiene behaviour, followed by ethnicity (*β* = − 0.13, *p* = 0.014) and risk perception (*β* = 0.13, *p* = 0.016). For social distancing behaviour, the determinants were self-efficacy (*β* = 0.25, *p* < 0.001), perceived advantages (*β* = 0.15, *p* = 0.022), trust in policy (*β* = 0.14, *p* = 0.026) and gender (*β* = − 0.14, *p =* 0.016). Regression models explained 40% hand hygiene and 25% social distancing variance.

**Conclusions:**

This study indicated that communications about effectiveness of hand hygiene and social distancing behaviours had been effective in terms of knowledge acquisition. However, in the light of likely second waves of COVID-19, attention to maintaining social distancing behaviour and improving hand hygiene behaviour may need to address more difficult areas of changing habits, overcoming time factors and building trust, as well as interventions to increase self-efficacy and address risk perception concerns.

**Supplementary Information:**

The online version contains supplementary material available at 10.1186/s12889-021-10461-0.

## Background

The Coronavirus disease 19 (COVID-19) pandemic of 2020, which started in Wuhan, China, in December, 2019 resulted in over 100 million confirmed cases and 2 million deaths globally by late January, 2021 [1]. Treatment options are limited and in the absence of a vaccine, until recently, mitigation by nonpharmaceutical interventions (NPIs) has gained prominence. Both the World Health Organisation (WHO) and governments of individual countries, including the United Kingdom (UK), have promoted health-related behaviours such as hand washing and social distancing to protect communities and individuals from transmission of the causative virus, severe acute respiratory syndrome coronavirus 2 (SARS-CoV-2) [2, 3]. In the UK, on 23rd March 2020, with UK cases of 5687 and 285 deaths [1], the government imposed a “lockdown” on people’s movements, accompanied by a high level communications exercise, including leaflet drops to all households [4]. The communications, which are ongoing at the time of writing, included information about restrictions such as social distancing, as well as handwashing and cough etiquette (hand hygiene). The degree to which such health behaviours are then practised or accepted is dependent not only on such communications, but is likely to be linked to factors such as knowledge of effectiveness/perceived efficacy of behaviours, and risk perception [5, 6]. It is recognised that gaining such behavioural insights during COVID-19 will be critical in ensuring that effective strategies are put into place to encourage compliance with recommended practices and manage transmission of the disease [7].

While COVID-19, like other respiratory pandemics, is typically seen as affecting the elderly and vulnerable [8], there are exceptions, as demonstrated by the 1918 Spanish flu pandemic and H1N1 swine flu which primarily impacted young adults [9, 10]. In addition, the evidence that COVID-19 can also be spread by individuals with minor symptoms as well as the asymptomatic [11, 12], suggests that the degree to which university students practice these protective behaviours, as well as the determinants behind compliance, is relevant. Certainly, for the H1N1 flu pandemic, Seale [13] emphasised the importance of university students carrying out protective behaviour. Many universities had already closed before lockdown commenced. However, understanding these behaviours in university students could ensure appropriate protective behaviours continue at home, and are in place for the safe re-opening of universities in the future. The value of surveys in assessing knowledge, attitudes and practices in respiratory illness pandemics to identify needs and guide policy, has been commented on by Hsu [14]. However, at the time of concept design for this study, there was a paucity of literature on determinants of protective behaviours during the COVID-19 pandemic, which had only been declared such on March 13, 2020. With no studies apparent in university students in the UK, it was paramount to fill in such a knowledge gap while the initial UK lockdown was in force and there was a significant impact on people’s lives.

The aim of the study was to investigate the effect of knowledge, socio-cognitive perceptions and demographic characteristics on protective behaviours such as hand hygiene and social distancing, in university students at the time of the COVID-19 pandemic. The objectives were to 1) Investigate the knowledge/socio-cognitive perceptions towards hand hygiene and social distancing; 2) Investigate which determinants explain hand hygiene and social distancing. Identification of determinants of hand hygiene and social distancing behaviours in UK university students may assist in determining interventions or policies to improve practice of these protective behaviours, if this is needed, and may also provide learnings for the general population. This may support provision of safer environments later in the pandemic, in future waves or in future pandemics.

## Methods

### Theoretical rationale

Bish and Michie [15] recommend the use of theoretical frameworks in studies of determinants of protective behaviours during pandemics. Although use of a theoretical framework is key, the scale of the impact of COVID-19 is unprecedented compared to previous pandemics in the last century and so a wide pool of potential determinants was considered for the current study based on literature, from theory or empirical studies.

Integrative models can be used to make sense of all these determinants of behaviour. The Integrated-Change (I-Change) model integrates a range of socio-cognitive theories to explain behavioural changes by looking at pre-motivational factors such as knowledge and risk perception, as well as motivational factors such as attitude, social norms and self-efficacy [16]. It also includes predisposing factors which can represent a wide range of behavioural, psychological, biological and environmental factors, and hence can provide a comprehensive framework for investigating a wide variety of potential determinants, including habit, time factors [17] and trust [18]. Therefore, the current study used I-Change as a theoretical framework.

### Study design

A cross-sectional design was used, incorporating an online survey, appropriate both in terms of lockdown restrictions during the COVID-19 pandemic as well as familiarity of university students with online surveys.

Protective behaviour outcome or dependent variables were the domains of hand hygiene, and social distancing behaviours. Predictor (independent) variables for the factors or determinants comprised domains of sociodemographic factors (e.g. gender, age, ethnicity), knowledge (of disease or effectiveness of behaviours), and socio-cognitive factors, both motivational/pre-motivational (e.g. risk perception, attitude, social support, self-efficacy), and predisposing (e.g. habit/automaticity, time factors, trust in authorities’ policies). The study was informed by the Strengthening the Reporting of Observational Studies in Epidemiology (STROBE) guidelines [19].

### Study setting and sampling

The survey was conducted online with paid participants recruited by Prolific Academic Ltd. [20], a crowdsourcing platform. Prolific identified 4250 potential participants from their standard pre-screening, who were currently residing in the UK at the end of the initial “lockdown” period of the COVID-19 pandemic, were currently undertaking a university course at undergraduate, graduate or postgraduate level (nearest match to study eligibility criteria of current UK university students) and were at least 18 years old. From the sample size calculations for multiple linear regression, a minimum sample size of 206 would detect a medium effect size of 0.1, with 80% power and α error probability of 5%. The targeted sample size was 300, to account for responders who were not eligible but passed the pre-screening/profiling criteria, or missed responses/questions or where there were incorrect assumptions for the sample size calculation. Pre-screened participants enrolled in the study on a first-come, first-served basis, after receipt of an email sent out by Prolific to a random subset of all potentially eligible participants.

### Questionnaire design

The questionnaire was adapted from existing surveys, primarily a WHO longitudinal survey on monitoring behavioural insights for use during the COVID-19 pandemic [7, 21]. A subset of the WHO questions was used to allow focus on support for the predictor and outcome variables relevant to this study and limit the questionnaire to an acceptable length. Adaption was also required to ensure consistency with the UK government communications of the time, and information likely to be known from the media or more formal sources such as the WHO.

Questions were selected to address the I-Change pre-motivational factors such as knowledge and risk perception, as well as motivational factors such as attitudes (advantages, disadvantages), social support and self-efficacy as appropriate for the different behaviours of hand hygiene and social distancing [16]. Additional predisposing factors were identified from the literature. Thus, hand hygiene questions included time factors and habit/automaticity, but not social support [17], while social distancing questions covered all 4 motivational factors from I-Change (advantages, disadvantages, social support, self-efficacy), but also perception of trust in government interventions [21]. Risk perception items and knowledge of the effectiveness of protective behaviours also reflect the work of Rubin [22] for designing a questionnaire on perceptions and behaviour during an influenza pandemic. The questionnaire consisted of 98 items from 28 questions, primarily multiple choice and Likert scales and can be found as Additional file 1. It took 10–15 min to complete. A data dictionary can be requested from the corresponding author.

Reliability of the composite scales in the questionnaire was determined using Cronbach’s alpha coefficient. Pallant [23] notes that although a Cronbach alpha coefficient should be above 0.7, with short scales of less than 10 items, low Cronbach’s values such as 0.5 are commonly found. Therefore, a threshold of 0.5 was used for scales. Due to the heightened time scales, formal content validity was not performed. However, as well as the questions being informed by previous surveys, a ‘Think aloud’ review by departmental colleagues was performed to increase confidence in validity. In addition, a pilot of 10 participants matching the inclusion criteria was carried out using the Prolific recruitment process and online platform before the main survey was launched, to check for any issues.

#### Demographics

Demographics included age, gender (male, female, other), student status (UK, International), health/life-science related course (Yes, No), and ethnicity (white, black, asian, chinese, mixed, other), all of which could influence protective behaviours, as reviewed by Bish and Michie [15] and assessed by Ergin [24] and Seale et al [13]. In addition, known exposure to infection (self or immediate social environment) was assessed [21].

#### Behaviour

The outcome or ‘dependent’ variables comprised the hand hygiene behaviour scale (α = 0.75) and the social distancing behaviour scale (α = 0.74). Scores for these were derived using three-point Likert scales of 8 and 10 questions respectively, based on a combination of current government guidance at the time [3], the WHO longitudinal survey [21] and, for handwashing, published surveys [17, 25, 26]. Options were “Always” (3 points), “Mostly” (1 point), or “Rarely/never” (hand hygiene) or “Rarely/mostly” (social distancing) (0 points). The mean scores were used, adjusted in the case of hand hygiene for participants not required to answer the question on handwashing after touching pets, where that was not relevant. Possible scores therefore ranged from zero to three. Due to the criticality of this score, the mean was also calculated for the 3 cases with individual item missing data in the social distancing behaviour, and thus in these cases the missing data could be considered imputed. Additional understanding of hand hygiene behaviour such as whether soap and water was used, if hands were washed for the minimum recommended 20 seconds, reasons for not washing hands, and whether handwashing had increased since the start of the pandemic, were also gathered.

#### Knowledge

Knowledge was divided into disease knowledge or that specific to the effectiveness of carrying out specific hand hygiene or social distancing behaviours. Disease knowledge comprised 4 questions, assessed by appropriate responses to identification of symptoms (“Related”, “Not related”, “Don’t know”), at risk groups (“Are at risk”, “Are not at risk”, “Don’t know”), treatment availability, and incubation period (both multiple choice). Knowledge of the effectiveness of the behaviours was assessed by 8 questions each as “Yes”, “No”, “Don’t know”. Correctness of answers was determined based on independently verifiable knowledge at the time of the survey [2, 3, 21], with correct answers awarded one point. Where there was lack of clarity or doubt for certain symptoms or risk factors in the public message of the time, “Don’t know” was an acceptable alternative. Examples of correct answers for risk groups were – diabetes “At risk”, children aged 1–5 “Not at risk”, pregnant women “Not at risk” or “Don’t know”. Knowledge scales were derived by adding correct answers, although for symptoms and risk factors, all items needed to be correct to gain a symptom or risk factor knowledge point. Total scores for each scale were determined, but as these scales consisted of multiple dimensions and could be considered an index, Cronbach’s α calculation was not performed [27].

#### Risk perception

Risk perception was assessed based on the perceived probability, susceptibility and severity components used in the WHO survey [21], and as defined by Brewer [28], but using a 9-point scale. The mean risk perception score for the three components was derived (α = 0.66), with possible scores of one to nine.

#### Other socio-cognitive constructs

The socio-cognitive constructs were derived from 3 or 4 statements for each construct, using 5-point Likert scales (1 – “Strongly disagree” to 5 – “strongly agree”) as in Additional file 2, Tables S4 and S5. The individual’s mean score was derived for each construct. Individual missing items resulted in very few (≤ 5) cases per construct with missing data, and therefore minimal impact on sample size and ability to detect an effect. Such cases were omitted from the analysis, rather than imputed, which could otherwise potentially introduce bias.

For hand hygiene behaviour the following socio-cognitive constructs were developed:

Attitude (Hand hygiene): In order to assess attitude towards carrying out the behaviour, perceived advantages such as whether the activities prevented infection in self or others, and disadvantages such as the effort involved or potential to hurt hands, were assessed. These were measured through 4 advantages items and 3 disadvantages statements, with the latter reversed coded to support an overall attitudes (advantages minus disadvantages [27]) scale with Cronbach α = 0.58, although mean inter-item correlation was 0.19. Greater reliability may have been possible through use of the advantages (α = 0.61, mean inter-item correlation 0.31) and disadvantages (α = 0.55, mean inter-item correlation 0.33) subscales.

Self-efficacy (Hand hygiene): Confidence in ability to carry out hand hygiene behaviour was assessed using 3 items (α = 0.62).

Habit: Habit or the related automaticity (α = 0.64) was assessed by statements such as feeling strange if they do not wash hands after using the toilet, washing hands before eating being performed automatically, washing hands or having a tissue ready without realising it.

Time: Assessment of time factors (α = 0.55) was by use of concepts such as washing hands after the toilet even when busy, believing that hand washing before food preparation takes too much time (reverse coded), and seeing washing hands with soap and water as quick and easy.

All social distancing socio-cognitive factors were assessed as 3 item constructs.

Attitude (social distancing): Attitude (α = 0.46) was represented by an advantages scale (α = 0.85) covering perception of protection for self, others and the National Health Service (NHS), minus disadvantages scale (α =0.48), which included perceptions of missing family/friends, job concerns or being bored (reverse coded). As the attitude scale did not demonstrate acceptable reliability, the subscales were used in association analysis. Additionally, as the disadvantages subscale also was below the internal acceptability threshold, a sensitivity analysis was performed using an item which was thought to best summarise the construct [29], in this case whether the respondent thought they would lose their job or that of someone close to them.

Social norms/support: Social support for carrying out social distancing included statements about family and friends avoiding crowds or social contacts, and a reverse coded item on being encouraged to meet against guidelines (α = 0.67).

Trust: The ‘trust’ construct was about perceptions of policies such as agreeing with restricting liberty rights, whether the decisions were fair, and whether they should be relaxed even while many new COVID-19 cases were appearing (α = 0.39). Due to the low alpha, the scale was re-assessed and two key single items used in association analysis, 1) fairness of decisions and 2) relaxation of restrictions (reverse coded).

Self-efficacy (Social distancing): Similar to hand hygiene behaviour, self-efficacy for social distancing behaviour involved confidence in ability to carry out the behaviour (α = 0.75).

Self-efficacy (Infection avoidance): Applicable to both behaviours, self-efficacy at the level of general infection avoidance was assessed from a 9-point scale from “Extremely difficult” to “Extremely easy”.

Behaviour: Perception of behaviour compliance was assessed by two ‘Yes/No’ questions to determine participants’ perception of whether they complied with guidance for the behaviours.

### Data collection

Data were collected using the online survey questionnaire in Additional file 1, developed using and hosted, by Bristol Online Survey. Data collection was carried out on 13 May, 2020, the first day after a 7 week initial ‘lockdown’ period in the UK, which had commenced 23rd March [30]. Attention check questions such as reversal of expected answers were included in the questionnaire to determine careless responding [31]. Participants were recompensed £1.40 through Prolific, for their time for completing the survey.

Confidentiality was ensured as participants did not provide any personally identifiable information. Participants provided electronic written informed consent via questions at the start of the questionnaire, before being able to continue with the survey.

### Data analysis

Data quality checks were carried out such as time to perform the survey, and data scrutinised for consistent single response on Likert scale across all categories, with the result that all 293 participants were included. Statistical analysis was performed using IBM Statistical Package for the Social Sciences (SPSS) v 26.

The first objective of the study, to investigate the socio-cognitive perceptions towards hand hygiene and social distancing, was addressed through descriptive statistics, primarily frequencies and percentages for behaviour, knowledge, risk perception, and other socio-cognitive perceptions. All scales were found to show other than a normal distribution by the Kolmogorov-Smirnov test, and therefore medians were used, although mean and standard deviation (SD) were also reported.

The second objective of the study, to investigate which determinants (such as knowledge and socio-cognitive perceptions) explain hand hygiene and social distancing, was addressed through inferential statistics, notably associations such as in bivariate correlations and multiple linear regression. Due to the distribution, non-parametric tests such as Spearman’s rank correlation co-efficient or Chi-squared analysis were used for testing associations between each behaviour (scale variable), and the potential determinants (scale, dichotomous or nominal variables). Where minimum cell count assumptions for Chi-squared were not met, Likelihood Ratio was used.

Hierarchical multiple linear regression was performed to develop an explanatory model to fit the data and assess the main predictors or determinants influencing the behaviour scales [32]. The model was performed as a hierarchical regression in 3 stages; an initial block of socio-demographic variables, as co-variates, some of which have been identified as determinants in previous studies [5, 33], a second block of potential predisposing (habit, time factors, trust), or pre-motivational (knowledge, risk perception) predictors identified previously in literature [17, 34, 35], followed by other potential motivational determinants (attitude, social support, self-efficacy) in a third stage. All potential determinants variables, including those not significant in bivariate correlation analysis, were therefore assessed in the model [32]. Where Cronbach alpha internal reliability for scales was < 0.5, individual subscales, or items were entered into the analysis [29, 36] as a sensitivity analysis, as well as assessed in the correlation analysis. Final parsimonious linear regression models of the determinants with the highest standardised *β* coefficients were also produced. Separate analysis by gender was also performed.

For the hand hygiene linear regression, assumptions for regression models including absence of collinearity were essentially met. However, for the social distancing regression model, a second dataset was used for linear regression modelling and correlations, due to the presence of 12 extreme outliers (1st quartile – 3*interquartile range (IQR), or 1st quartile – 1.5* IQR for > 4 constructs (or 3rd quartile + 1.5*IQR equivalent)) [37] which otherwise would have led to contravention of regression assumptions. Sample size was thus reduced to a maximum of 281 for social distancing inferential statistical analyses. To ensure robust regression modelling in the presence of scales which may have diverged from normal distribution, bootstrapping of 1000 samples for calculation of bias-corrected and accelerated (BCa) 95% confidence intervals (CI) and significance, was used.

Correlation matrices for all general and hand hygiene related variables, as well all general and social distancing related variables, were also created to determine if there were any strong relationships between individual predictor variables which may have then impacted the multiple linear regression findings. Post hoc correlation and linear regression analysis was performed using individual components of risk perception (probability, susceptibility, severity), to further investigate associations between these aspects of risk perception, and hand hygiene and social distancing behaviours. Where key determinants from the literature did not show significant correlation or appear significant in the linear regression models in this study, post hoc analysis was undertaken by use of dichotomised variables (such as for ethnicity – white/minorities), or item by item correlation (knowledge of effectiveness of specific behaviour versus the matching behaviour) to see whether relationships could then be identified.

Statistical significance was set at *p* < 0.05, 2-tailed, and 95% confidence intervals were reported.

## Results

### Sample characteristics

Surveys were sent out to 315 pre-screened participants by Prolific, and responses received by 305, of whom 293 met the inclusion criteria of currently attending a UK university, and who were eligible for analysis. Female students comprised 65.2%. The median age was 22 (IQR: 20, 25) with a range of 18 to 52. Of the 293 participants, 23.5% identified as a minority ethnicity. Table 1 shows the socio-demographic characteristics of the participants.

**Table 1:** Socio-demographic characteristics of the participants

Characteristic	***N*** = 293 (%)
**Age (*n*** **= 293)**	
18–25	215 (73.4)
> 25	78 (26.6)
**Gender (*n*** **= 290)**	
Male	100 (34.5)
Female	189 (65.2)
Other	1 (0.3)
**Student Status (*n*** **= 289)**	
UK	250 (86.5)
International	39 (13.5)
**Health-related course (*n*** **= 290*)***	
Yes	74 (25.5)
No	216 (74.5)
**Ethnicity (*n*** **= 293)**	
White	224 (76.5)
Black	14 (4.8)
Asian	32 (10.9)
Chinese	3 (1.0)
Mixed	15 (5.1)
Other	5 (1.7)
**Infection exposure (self or peers) (*n*** **= 291)**	
Yes	94 (32.3)
No	197 (67.7)

### Behaviours

For hand hygiene behaviour, 42.0% of participants reported that they carried out every activity “Mostly” or “Always” with only 8.5% reporting “Always”. Washing hands before touching the face followed by after touching animals, handling money/public equipment, and coughing/sneezing, were the activities least likely to be practised, with 44.0% of participants “Rarely/never” washing their hands before touching their face (Additional file 2, Table S1). Only 1.4% reported rarely or never washing hands after toilet use. In terms of other features of hand hygiene behaviour, 98.6% of participants reported washing hands with soap and water (or anti-bacterial hand gel), and 74.7% for at least 20 seconds. Most participants (88.0%) have increased their frequency or quality of handwashing since before the pandemic. The main reasons given for not washing hands were “Not used to it” (23%) and “Not enough time” (14%).

For social distancing behaviour, unlike in hand hygiene behaviour, a very high proportion (88.9%) reported that they carried out every social distancing activity “Mostly” or “Always”, with 47.6% reporting “Always”. “Shopping only for basic necessities, as infrequently as possible” was the activity with the lowest compliance (“Always”: 70.0%), and “Not meeting in groups” had the highest compliance (“Always”: 94.7%) (Additional file 2, Table S2).

### Knowledge and socio-cognitive perceptions

Almost all respondents (95.9%), correctly reported that there was no treatment or vaccine in the UK at the time of the survey, with 86.7% correctly reporting that the maximum incubation period was 14 days. Additionally, 90.7% identified all 3 key symptoms (fever, cough, shortness of breath), but understanding of other symptoms was less clear (Additional file 2, Fig. S1A). Similarly, while 98.6% identified the elderly as at risk of severe disease, their ability to discern risk of other groups was mixed (Additional file 2, Fig. S1B).

Students’ perceptions of severity, susceptibility and probability of contracting COVID-19, tended to be in the lower areas of the scales (Additional file 2, Table S3). Sixty nine percent of students reported that it would be easy to extremely easy to avoid infection with the virus.

#### Hand hygiene

Correct knowledge of at least 7 of 8 hand hygiene activities effective in preventing or not preventing infection with SARS-CoV-2 was identified by 90.7% of students. Perception of positive compliance with the guidance on hand hygiene behaviour was reported by 95.9%.

Responses for hand hygiene socio-cognitive perceptions regarding motivational factors such as attitudes (perceived advantages and disadvantages) and self-efficacy, and preceding factors such as perceived time factors and habit/automaticity are shown in Additional file 2, Table S4.

#### Social distancing

Correct knowledge of at least 9 of 10 hand social distancing activities effective in preventing or not preventing infection with SARS-CoV-2 was identified by 93.5% of students. Knowledge of the need to stay at home for 7 days or until symptoms stop, if COVID-19 fever and/or cough symptoms develop, was the item most likely to be assessed incorrectly, with 10.7% reporting “No”. Perception of positive compliance with social distancing guidance was reported by 96.2%.

Responses for social distancing socio-cognitive perceptions regarding motivational factors such as attitudes (perceived advantages and disadvantages), social support and self-efficacy, and preceding factors such as trust are shown in Additional file 2, Table S5.

### Associations between knowledge and socio-cognitive perceptions, and behaviours

#### Hand hygiene behaviour correlations

From bivariate analysis, as shown in Table 2, there was a significant positive correlation at *p* ≤ 0.001 between hand hygiene behaviour and attitude, time factors, habit and self-efficacy (all medium effect size, *r*_*s*_ > 0.3) while effect size for the correlation between hand hygiene behaviour and attitude or risk perception was small but significant. Gender showed a small but significant negative correlation, with lower reported hand hygiene behaviour in males than in females.

**Table 2:** Correlation between factors (demographic, knowledge, socio-cognitive) and hand hygiene behaviour

Factor	***N***	Mean (SD)	Median (IQR) ^**a**^	Correlation Hand Hygiene Behaviour	
				Spearman’s ***r***_***s***_ (2-tailed)[95% BCa CI]^**b**^	***p***
Hand hygiene behaviour score	293	1.78 (0.69)	1.75 (1)	–	–
Age	293	22.58 (5.80)	22 (5)	0.01 [− 0.11, 0.12]	0.857
Gender (F/M)	289	N/A	N/A	−0.23 [− 0.33, − 0.12]	**< 0.001**
Ethnicity (Minorities/White)	293	N/A	N/A	−0.08 [− 0.20, 0.04]	0.152
Student home/Intl status	289	N/A	N/A	−0.07 [− 0.18, 0.043]	0.222
Health course	290	N/A	N/A	0.04 [−0.08, 0.16]	0.480
Exposure	291	N/A	N/A	0.11 [−0.004, 0.23]	0.064
Disease knowledge	287	2.52 (0.75)	3.00 (1.0)	0.10 [−0.02, 0.22]	0.086
Risk perception	293	4.31 (1.55)	4.33 (2.16)	0.20 [0.10, 0.29]	**0.001**
Self-efficacy – infection avoidance	293	6.12 (1.87)	6.0 (2.0)	−0.08 [−0.20, 0.04]	0.158
Hand hygiene effectiveness knowledge	290	7.53 (0.70)	8.0 (1.0)	−0.07 [0.019, 0.07]	0.239
Advantages	293	4.16 (0.54)	4.25 (0.5)	0.29 [0.19, 0.39]	**< 0.001**
Disadvantages (reversed)	292	4.06 (0.77)	3.67 (1.33)	0.19 [0.06, 0.31]	**0.001**
Attitudes	292	2.22 (1.02)	2.33 (2.0)	0.26 [0.15, 0.37]	**< 0.001**
Time factor	293	4.24 (0.68)	4.0 (1.0)	0.49 [0.40, 0.58]	**< 0.001**
Habit	290	3.74 (0.85)	4.0 (1.0)	0.58 [0.50, 0.66]	**< 0.001**
Self-efficacy – Hand hygiene	293	4.47 (0.58)	4.67 (1.0)	0.38 [0.27, 0.48]	**< 0.001**

*Abbreviations: N/A* not applicable, *BCa CI* Bias corrected accelerated confidence intervals, *SD* standard deviation, *IQR* interquartile range Bold: significance *p* < 0.05

^a^ Median (IQR) reported as based on the Kolmogorov-Smirnov test for normality, variables were not normally distributed

^b^ 95% BCa CI from 1000 bootstrap samples

Perception of compliance with hand hygiene guidance was significantly associated with hand hygiene behaviour, *r*_*s*_ = 0.26, *95%* BCa CI [0.16, 0.35], *n* = 292, *p* < 0.001. Hand hygiene behaviour did not differ significantly with ethnicity in analysis of all ethnic groups, Likelihood Ratio (1180) = 158.06, *p* = 0.879.

A correlation matrix for 18 general and hand hygiene related variables can be found in Table S6 in Additional file 2.

#### Social distancing behaviour correlations

As shown in Table 3, there was a significant positive correlation between social distancing behaviour and advantages, social support self-efficacy for social distancing, aspects of trust in the restrictive policies (all *p* < 0.01), and self-efficacy for infection avoidance (*p* < 0.05). For self-efficacy for social distancing, correlation reached a medium effect size (*r*_*s*_ = 0.43). Gender showed a small but significant negative correlation with males showing lower reported behaviour than females, as did white ethnicity compared to minorities.

**Table 3:** Correlation between factors (demographic, knowledge, socio-cognitive) and social distancing behaviour

Factor	***N***	Mean (SD)	Median (IQR) ^**a**^	Correlation Social Distancing Behaviour	
				Spearman’s ***r***_***s***_ (2-tailed) [95% BCa CI]^**b**^	***p***
Social distancing behaviour score	281	2.60 (0.63)	2.75 (0.5)	–	–
Age	281	23.53 (5.75)	22 (5)	0.03 [−0.09, 0.15]	0.658
Gender (F/M)	278	N/A	N/A	−0.13 [− 0.26, − 0.01]	**0.026**
Ethnicity (Minorities/White)	281	N/A	N/A	−0.12 [− 0.23, 0.01]	**0.026**
Student home/Intl status	278	N/A	N/A	−0.03 [− 0.16, 0.09]	0.573
Health course	278	N/A	N/A	0.02 [−0.10, 0.14]	0.732
Exposure	279	N/A	N/A	0.014 [−0.10, 0.11]	0.814
Knowledge – COVID-19	275	2.53 (0.75)	3.0 (1.0)	0.06 [−0.06, 0.19]	0.293
Risk perception	281	4.31 (1.56)	4.33 (2.0)	0.07 [−0.04, 0.19]	0.217
Self-efficacy – avoidance of infection	281	6.14 (1.86)	6.0 (2.0)	0.12 [0.01, 0.24]	**0.047**
Social distancing effectiveness knowledge	277	9.66 (0.72)	10.0 (0)	0.11 [−0.02, 0.22]	0.075
Attitude^c^	277	0.87 (0.95)	1.0 (1.0)	0.16 [0.05, 0.27]	**0.009**
Advantages	280	4.61 (0.52)	5.00 (0.92)	0.26 [0.13, 0.38]	**< 0.001**
Disadvantages (reversed) ^c^	278	2.26 (0.81)	2.33 (1.33)	0.02 [−0.11, 0.16]	0.746
Social support	276	4.40 (0.65)	4.67 (1.0)	0.28 [0.15, 0.39]	**< 0.001**
Trust^d^	280	3.89 (0.71)	4.0 (1.0)	0.28 [0.16, 0.39]	**< 0.001**
Self-efficacy – social distance	280	4.39 (0.66)	4.67 (1.0)	0.43 [0.32, 0.52]	**< 0.001**

*Abbreviations*: *N/A* not applicable, *BCa CI* Bias corrected accelerated confidence intervals ,*SD* standard deviation; IQR, interquartile range Bold: significance *p* < 0.05

^a^ Median (IQR) reported as based on the Kolmogorov-Smirnov test for normality, variables were not normally distributed

^b^ 95% BCa CI from 1000 bootstrap samples

^c^ Cronbach α for Attitude < 0.5 so subscales (advantages and disadvantages) analysed. Cronbach α for Disadvantages subscale < 0.5 so key representative item analysed “I think I will lose my job (or have lost my job and will not be able to find other work)” – reversed - correlation with social distancing behaviour: *r*_*s*_ = 0.09 [− 0.04, 0.21], *p* = 0.153

^d^ Cronbach α for Trust < 0.5, so two key items analysed “I think the decisions that have been made to reduce the spread of the novel coronavirus are fair”- correlation with social distancing behaviour: *r*_*s*_ = 0.21 [0.10, 0.33], *p* = 0.001; “I think the authorities should relax the restrictions even if there are still many new cases of COVID-19 emerging” – reversed - correlation with social distancing behaviour: *r*_*s*_ = 0.24 [0.12, 0.36], *p* < 0.001

Perception of compliance with social distancing guidance was significantly associated with social distancing behaviour, *r*_*s*_ = 0.27, 95% BCa CI [0.18, 0.36], *n* = 276, *p* < 0.001. In contrast to the correlation shown for ethnicity as a dichotomous variable (Table 3), social distancing behaviour did not differ significantly with ethnicity in analysis of all ethnic groups, Likelihood Ratio (1, 75) = 56.82, *p* = 0.942.

A correlation matrix for all 17 general and social distancing related variables can be found in Table S7 in Additional file 2. A significant positive correlation was found between social distancing behaviour score and hand hygiene behaviour: *r*_*s*_ = 0.319, 95% BCa CI [0.21, 0.42], *p* < 0.001, *n* = 281.

#### Components of risk perception

A more detailed analysis of the components of risk perception showed that perceived susceptibility and severity had a greater association (larger *r*_*s*_ coefficient) with hand hygiene behaviour than perceived probability, although all retained significance (Table 4). Analysis of risk perception components did not identify a component which was significantly correlated with social distancing behaviour.

**Table 4:** Correlation between perception of probability, susceptibility and severity of infection and protective behaviours

Factor	***N***	Mean (SD)	Median (IQR) ^a^	Spearman’s correlation ***r***_***s***_ (2-tailed) [95% BCa CI] ^b^	***p***
**Hand hygiene behaviour**					
Probability	293	4.41 (1.96)	4.0 (3.0)	0.13 (0.02, 0,23]	**0.031**
Susceptibility	293	4.41 (1.97)	4.0 (3.0)	0.17 [0.06, 0.26]	**0.005**
Severity	293	4.13 (2.12)	4.0 (4.0)	0.18 [0.07,0.28]	**0.002**
**Social distancing behaviour**					
Probability	281	4.44 (1.97)	4.0 (3.0)	0.006 [−0.10, 0.11]	0.916
Susceptibility	281	4.41 (1.97)	4.0 (3.0)	0.03 [−0.08, 0.15]	0.613
Severity	281	4.07 (2.09)	4.0 (4.0)	0.107 [−0.01, 0.22]	0.074

*Abbreviations*: *BCa CI*, Bias corrected accelerated confidence intervals, *SD* standard deviation, *IQR* interquartile range. Bold: significance *p* < 0.05

^a^ Median (IQR) reported as based on the Kolmogorov-Smirnov test for normality, variables were not normally distributed

^b^ 95% BCa CI from 1000 bootstrap samples

### Linear regression models explaining behaviours

Multiple linear regression identified which I-Change constructs predominantly explained the self-reported hand hygiene behaviours (Table 5). The model was performed as a hierarchical regression in 3 stages; an initial block of socio-demographic variables, a second block of potential predictors identified previously in literature and equivalent to I-Change predisposing and pre-motivational factors, followed by other potential motivational determinants in a third block, resulting in 3 models. Each model also retained the potential factors from the previous model. Predisposing factors of habit (*β* = 0.39) and then time factors (*β* = 0.28) were the greatest contributors to unique variance in hand hygiene behaviour (*p* = 0.001). Risk perception (*β* = 0.13) and ethnicity (*β* = − 0.13) added additional contributions, as did gender (*β* = − 0.09), although the latter did not attain significance with the use of robust regression methodology (*p* = 0.068*)*. A parsimonious linear regression model based on these five determinants explained 40% of the variation in hand hygiene behaviour *R*^*2*^ = 0.41, adjusted *R*^*2*^ = 0.40, *F* (5,280) = 39.3, *p* < 0.001. In separate gender specific analyses, time, habit and ethnicity were retained as significant in the model for females, but not risk perception. For males, only habit was significant in the model. Probability of infection, susceptibility and severity as individual aspects of risk perception, did not show a significant effect. Other factors such as attitude, self-efficacy and knowledge of the disease or effectiveness of hand hygiene behaviours did not separately contribute to the model.

**Table 5:** Multiple linear regression for demographic, predisposing, pre-motivational, motivational factors predicting hand hygiene behaviour

	Model 1: Sociodemographic		Model 2: Socio-demographic, predisposing, and pre-motivational		Model 3: Socio-demographic, predisposing, pre-motivational and motivational		
	Standardized Coefficients	*p*	Standardized Coefficients	*p*	Unstandardized Coefficients	Standardized Coefficients	*p* ^a^
	Beta		Beta		B (SE)	Beta (95% BCa CI) ^a^	
(Constant)		< 0.001		0.954	−0.1 (0.52)		
**Socio-demographic**							
Gender (Female/Male)	−0.23	**< 0.001**	−0.09	0.055	−0.13 (0.07)	− 0.09 (− 0.2, 0.01)	0.068
Age	0.02	0.801	−0.04	0.430	−0.004 (0.01)	−0.04 (− 0.15, 0.07)	0.474
Student Status (UK/International)	−0.09	0.143	−0.01	0.803	−0.01 (0.1)	− 0.01 (− 0.1, 0.08)	0.880
Health Course	− 0.01	0.824	0.00	0.987	−0.005 (0.08)	0 (−0.1, 0.1)	0.944
Ethnicity (Minorities/White)	−0.17	**0.007**	−0.13	**0.009**	−0.22 (0.08)	−0.13 (− 0.24, − 0.02)	**0.014**
Infection exposure	0.08	0.191	0.01	0.818	0.01 (0.07)	0.01 (−0.1, 0.12)	0.896
**Predisposing factors**							
Habit			0.40	**< 0.001**	0.31 (0.05)	0.39 (0.26, 0.51)	**0.001**
Time factors			0.28	**< 0.001**	0.28 (0.07)	0.28 (0.14, 0.42)	**0.001**
**Pre-motivational factors**							
Disease knowledge			0.00	0.941	0.004 (0.04)	0 (−0.09, 0.11)	0.930
Hand hygiene effectiveness knowledge			−0.07	0.146	−0.06 (0.05)	− 0.07 (− 0.16, 0.03)	0.133
Risk perception			0.14	**0.003**	0.06 (0.02)	0.13 (0.02, 0.24)	**0.016**
**Motivational factors**							
Self-efficacy- infection avoidance					−0.01 (0.02)	−0.02 (− 0.13, 0.08)	0.722
Attitude					−0.02 (0.04)	− 0.04 (− 0.16, 0.11)	0.562
Self-efficacy - hand hygiene					0.04 (0.07)	0.03 (−0.09, 0.14)	0.633

*Abbreviations*: *BCa CI* Bias corrected accelerated confidence intervals, *SE* standard error. Bold: significance *p* < 0.05

^a^ Model 3 was calculated using 1000 bootstrap samples for robustness, for 95% BCa CI and *p* values

Model 1: R^2^ = 0.082, adjusted R^2^ = 0.061; Model 2: R^2^ = 0.452, adjusted R^2^ = 0.429, △ R^2^ *p* < 0.001; Model 3: R^2^ = 0.453, adjusted R^2^ = 0.423, △ R^2^ *p* = 0.866

For self-reported social distancing behaviours, multiple linear regression also identified I-Change constructs which significantly explained behaviour (Table 6). The model was also developed using the same 3 stages as the hand hygiene model (block 1: socio-demographic variables; block 2: predisposing and pre-motivational factors from the literature; block 3: motivational determinants) to produce 3 models. Subscales of attitude (advantages, disadvantages) were reported due to the low internal reliability of the attitude scale. The greatest contributor to unique variance was self-efficacy for the social distancing behaviour (*β* = 0.24, *p* = 0.001), followed by trust (*β* = 0.22, *p* = 0.003). Gender (*β* = − 0.14) and perceived advantages (*β* = 0.14) also contributed to the variance although perceived advantages did not reach significance with the use of robust regression methodology (*p* = 0.073). Potential effects of ethnicity apparent in model 2, were lost in model 3 with the addition of other factors which contributed to unique variance. When the trust scale was replaced with two key items of fairness and (lack of) trust in a loosening lockdown policy, and disadvantages scale replaced with one representative item of job concerns, due to the low measure of reliability for the trust and disadvantages scales, the significant explanatory determinants were self-efficacy (*β* = 0.25, *p* < 0.001), perceived advantages (*β* = 0.15, *p* = 0.022), trust in policy (*β* = 0.14, *p* = 0.026) and gender (*β* = − 0.14, *p =* 0.016). A parsimonious model of only the 4 main determinants explained 25% of the variation in social distancing behaviour *R*^*2*^ = 0.26, adjusted *R*^*2*^ = 0.25, *F* (4,270) = 23.5, *p* < 0.001, with all 4 determinants providing a significant contribution (self-efficacy and trust both *p* < 0.001; advantages *p* = 0.016; gender *p* = 0.025). Separate analysis by gender resulted in retention of significance for self-efficacy and trust in females but no significant variables for males. Probability of infection, susceptibility and severity as individual aspects of risk perception, or risk perception as a combined construct, did not show a significant effect. Potential factors such as social support and knowledge of the disease or of effectiveness of social distancing behaviour also did not separately contribute to the model.

**Table 6:** Multiple linear regression for demographic, predisposing, pre-motivational, motivational factors predicting social distancing behaviour

	Model 1: Sociodemographic		Model 2: Socio-demographic, predisposing, and pre-motivational		Model 3: Socio-demographic, predisposing, pre-motivational and motivational		
	Standardized Coefficients	*p*	Standardized Coefficients	*p*	Unstandardized Coefficients	Standardized Coefficients	*p* ^a^
	Beta		Beta		B (SE)	Beta (95% BCa CI) ^a^	
(Constant)		< 0.001		< 0.001	0.69 (0.4)		0.189
**Socio-Demographic**							
Gender (Female/Male)	−0.186	**0.004**	−0.140	**0.020**	−0.13 (0.05)	−0.14 (− 0.28, − 0.02)	**0.032**
Age	−0.032	0.609	−0.022	0.717	0.0 (0.004)	0.004 (−0.11, 0.12)	0.941
Student Status (UK/International)	−0.040	0.533	−0.017	0.771	−0.02 (0.07)	−0.02 (− 0.15, 0.1)	0.778
Health Course	−0.010	0.876	−0.018	0.758	−0.02 (0.06)	− 0.02 (− 0.12, 0.08)	0.744
Ethnicity (Minorities/White)	− 0.092	0.151	− 0.137	**0.024**	− 0.08 (0.06)	−0.08 (− 0.19, 0.03)	0.173
Infection exposure	−0.030	0.637	−0.068	0.264	−0.02 (0.05)	− 0.02 (− 0.14, 0.08)	0.713
**Predisposing and Pre-motivational factors**							
Trust			0.335	**< 0.001**	0.13 (0.04)	0.22 (0.09, 0.35)	**0.003**
Disease knowledge			0.061	0.309	0.03 (0.03)	0.06 (−0.05, 0.15)	0.296
Social distancing effectiveness knowledge			0.076	0.213	−0.02 (0.04)	−0.03 (− 0.2, 0.13)	0.728
Risk perception			0.037	0.546	0.01 (0.02)	0.05 (−0.07, 0.17)	0.409
**Motivational factors**							
Self-efficacy- infection avoidance					0.01 (0.01)	0.05 (−0.08, 0.19)	0.422
Advantages					0.12 (0.06)	0.14 (−0.02, 0.28)	0.073
Disadvantages (reversed)					0.02 (0.03)	0.03 (−0.09, 0.16)	0.612
Social support					0.06 (0.04)	0.1 (−0.03, 0.23)	0.175
Self-efficacy social distancing					0.16 (0.04)	0.24 (0.12, 0.35)	**0.001**

*Abbreviations*: *BCa CI* Bias corrected accelerated confidence intervals, *SE* standard error. Bold: significance *p* < 0.05

^a^ Model 3 was calculated using 1000 bootstrap samples for robustness, for 95% BCa CI and *p* values

Model 1: R^2^ = 0.043, adjusted R^2^ = 0.02; Model 2: R^2^ = 0.178, adjusted R^2^ = 0.145, △ R^2^ *p* < 0.001; Model 3: R^2^ = 0.280, adjusted R^2^ = 0.235, △ R^2^ *p* < 0.001

In post hoc analysis undertaken to investigate why perceived effectiveness of hand hygiene or social distancing behaviours did not show significant correlation or contribution to the linear regression models for the related behaviours, Chi-squared tests were performed on individual items from the related knowledge and behaviour scales or composite questions. For the 4 hand hygiene item behaviours which could be tested, only “sneeze or cough into tissue or sleeve” showed a significant association between perceived/knowledge of effectiveness and this behaviour (Likelihood Ratio (1,2) = 7.78, *p* = 0.02). Within the 8 social distancing items, only “Staying at home when sick” (Likelihood Ratio (1,2) = 6.01, *p* = 0.049) and “Limiting outside exercise to once a day and alone or with household members” (Likelihood Ratio (1,2) = 7.63, *p* = 0.022), showed significant associations. Most extreme outliers of very low social distancing behaviour scores removed from the social distancing dataset, showed inconsistencies in terms of high levels of knowledge about effectiveness of social distancing behaviour, positive perception of compliance and positive attitudes and self-efficacy for social distancing behaviours, validating their exclusion.

## Discussion

This study aimed to investigate the effect of knowledge, socio-cognitive perceptions and demographic characteristics on protective behaviours such as hand hygiene and social distancing, in UK university students at the time of the COVID-19 pandemic, and was carried out at the end of the first lockdown period in May, 2020. The study still appears to be the first study examining the determinants of protective behaviours in university students in Western countries during the COVID-19 pandemic. Two studies comprising medical students in Iran [38] and university students in Pakistan [39] have since been identified but without specifically describing both hygiene and social distancing activities, and with few determinants analysed. Studies in general populations or adolescents have been noted in the US, UK, and Australia [40–43], as well as studies in Asia. At the time of writing, there is no widespread effective treatment or vaccine, and non-pharmaceutical interventions, including protective behaviours such as hand hygiene and social distancing remain key to reducing transmission of the causative SARS-CoV-2 virus, and therefore the impact of the pandemic. While young adults, such as university students are usually deemed low risk for severe disease, they may still be sources of transmission. In the REal-time Assessment of Community Transmission (REACT) study of community transmission in the UK, carried out May, 2020, a similar time frame to this survey, it was the 18–24 year old age group who had the highest positivity rates and 69% of those positive were asymptomatic [44]. Asymptomatic transmission of SARS-Cov-2 from young people has been noted in the literature [45].

Although Bish and Michie [15] recommend the use of theoretical frameworks in studies of determinants of protective behaviours during pandemics, they note that few studies in their systematic review utilised such frameworks. Where a framework was used, the Health Belief Model (HBM) and Theory of Planned Behaviour (TPB) were the most common. More recently, Kupfer [46], linked hand hygiene behaviour determinants to the Theory of Interpersonal Behaviour, although this was in a non-pandemic environment. Aunger [17] developed a Behaviour Determination Model to explain the determinants of handwashing more generally, while Protection Motivation Theory has now been used for a study of preventative behaviours in healthcare workers in Iran during the COVID-19 pandemic [47]. Yet determinants identified as linking to the protective behaviours of interest, whether during a pandemic or more generally, and whether under a theoretical framework or outside, vary greatly, depending on the population. In university students, in previous pandemics, determinants have included risk perception (whether perceived severity, susceptibility, or concern/anxiety about the possibility of contracting the infection [48–50]), gender [48], perceived effectiveness of the measures [48, 51], course studied [52], country of birth [34] and self-efficacy and social norms [51]. Studies of the general population or outside pandemics add in knowledge, attitude and age [53], trust [54], ethnicity [29], habit and time factors [17].

### Investigation of knowledge/socio-cognitive perception towards hand hygiene and social distancing

To provide context for the knowledge/socio-cognitive perceptions for these protective behaviours, a comparison between the practice of the two behaviours and comparisons with frequency of the behaviours in other studies and pandemics is helpful. This may also identify if there is a greater need for interventions in one of the behaviours or particular activities within a behaviour. Participants reported increased handwashing (88%) during the pandemic as has been found in other studies of university students [38, 39, 49], and this exceeded increases in a general population in Australia during the initial stages of the COVID-19 pandemic [42] or the UK H1N1 outbreak [29]. Both the latter UK survey and this survey occurred after major government advertising campaigns, including leaflet drops, suggesting that in the current environment, the government information campaign has been particularly effective, as was also found in South Korea during the H1N1 pandemic [48]. However, there was only a moderate level of consistent (“Mostly” or “Always”) practice across all 8 hand hygiene activities (42.0%). While differences in the questions may affect comparability to other studies, this is still well within the wide range found in other studies, looking at handwashing alone or a broader range of hygiene behaviours [39, 55, 56]. Others have also found that specific hand hygiene behaviours such as face /mouth/nose touching may be particularly difficult [52].

In contrast to hand hygiene practice, a much greater proportion of study participants (88.9%) reported carrying out all eight social distancing behaviours. This contrasts with other studies where practice of social distancing measures was less than that of hygiene measure [42, 49]. This may reflect the strength of the government information campaign, although this aspect was beyond the scope of the current study. However, this high level of compliance gives less scope for analysis of determinants.

Knowledge and risk perception can be categorized as pre-motivational factors for behaviour within the wider I-Change model [16]. Student participants reported moderate to high levels of knowledge of the disease in terms of treatment, incubation period, symptoms and those most at risk, knowledge of all of which can impact behaviour. In particular, knowledge of all three key symptoms, and lack of treatment and vaccines was high which could potentially impact behaviours. As in the WHO survey [21], but in contrast to other studies [39, 57, 58], this study did not assess knowledge of transmission methods, because of uncertainties in the general public domain at the time about modes of transmission. In addition, participants exhibited high levels of knowledge of the effectiveness of hand hygiene and social distancing measures in preventing infection, with 90.7% and 93.5% respectively correctly identifying all or all but one measure.

Most students did not have high scores on the risk perception scale with a maximum score of 9 (mean 4.31, SD 1.55), in terms of perceived probability, susceptibility and severity, as might be expected for a younger population [38], although a study in Pakistan reported high levels of perceived risk regarding COVID-19 even in university students [39]. The latter may be due to differences in the impact of COVID-19 in the population as a whole in some countries with greater health and social system challenges than the UK. Risk perception was assessed through 3 constructs of probability (likelihood of becoming infected generally), susceptibility (perception of being more susceptible to becoming infected), and severity, as described by Brewer [28] and recommended by the WHO COVID-19 survey [21]. This differs from the 2 factor susceptibility and severity or the more general ‘perceived threat’ or concern about becoming infected seen in other determinant studies [50], and therefore comparison of risk perception is on a more generalised basis.

In terms of the main motivational or socio-cognitive factors from the I-Change model, overall study participants were positive about the perceived advantages of the behaviours, with most having confidence in their ability to practice them, and to avoid infection generally (self-efficacy). Social support for social distancing was mainly present. Perceived disadvantages were more complex, as while for hand hygiene, they mirrored the advantages, with social distancing there was no clear pattern.

The I-Change model has flexibility to support wider factors including predisposing factors, which in this study, allowed inclusion of habit and time factors from Aunger’s classification for handwashing [17], and trust which was present in the WHO survey and appears in other studies [18, 54]. Both habit and time factor statement responses showed variability in this sample although were skewed towards agreement. Trust was more balanced between agreement and disagreement. These findings in the full sample supported assessment of relationships with the two behaviours.

### Investigation of the determinants explaining hand hygiene and social distancing behaviours

Gender, age, ethnicity, education, perceived susceptibility and severity, as well as belief in effectiveness of recommended behaviours have all been shown to be associated with adoption of protective behaviours including handwashing and social distancing, most frequently described as avoidance behaviour, during pandemics [15]. In the 2 studies of university populations during the COVID-19 pandemic, only risk perception [38] and gender and economic category [39] have been demonstrated as determinants of protective behaviour. However, in university students in previous pandemics, social norms and self-efficacy [51], perceived threat or concern [49], empathic responding [50], course [52] and country of birth [34] have been additionally identified as determinants.

In this study, bivariate analysis also showed significant correlation for both hygiene and social distancing behaviours, with a certain determinants or factors, including gender, attitude (advantages) and self-efficacy. Course or UK/International status (analogous to Van’s country of birth [34]) did not show significant correlation with the two behaviour scales, even though those studying a health related course might be expected to be more aware of the need for hand hygiene. There was also no correlation between course and disease or hand hygiene knowledge (Additional file 2, Table S6), although knowledge is not equivalent to awareness in the I-Change model [16]. Not all the potential determinants identified in the literature using a variety of theories were collected in this survey, as the study was designed to cover a broad range of potential determinants which could impact design of interventions or public health communications, rather than focus on specifics such as empathic responding or anxiety. However, it was in examining associations specific to each of the behaviour scales, and subsequently carrying out multivariate analysis through multiple linear regressions enabling relationships between covariates to be revealed, that differences from previous literature started to emerge.

#### Determinants of hand hygiene behaviour

For hand hygiene, two additional potential determinants to standard I-Change Model socio-cognitive factors, were included from Aunger’s Behavioural Determination Model [17], which applied to hygiene measures in the context of understanding determinants effecting transmission of infectious disease. These were habit (automaticity, as a reactivity factor) and time factors (‘being busy’, as a situational factor) which had the greatest effect in the Aunger model for handwashing. They were deemed ‘predisposing factors’ in the context of the I-Change model [16]. In bivariate analysis using Spearman’s correlation, habit and time yielded large effect sizes (*r*_*s*_ ~ > 0.5) while the standard socio-cognitive factors of self-efficacy, attitude/advantages had medium effect sizes(*r*_*s*_ ~ > 0.3) and risk perception and the gender demographic variable effect sizes were small (*r*_*s*_ > 0.1, males having lower hand hygiene behaviour than females), although all were *p* ≤ 0.001. Ethnicity and knowledge, whether of the disease or effectiveness of hand hygiene measures did not show correlation with hand hygiene behaviour.

In multivariate analysis, the multiple linear regression confirmed that habit and time factors had the greatest contribution to the hand hygiene behaviours scores. The more integrative nature of I-Change supported the inclusion of other such predisposing factors from Aunger’s theoretical framework for handwashing [17]. These two determinants, habit and time factors, do not appear to have been previously identified in pandemic situations, either in the general population or in university students, and may need different strategies than knowledge based public health communications to effect behavioural change. Participants with higher risk perception, as identified in a broader sense by other university student surveys in previous pandemics [48–50], or the general population in the COVID-19 pandemic [42, 59, 60], were also more likely to perform hygiene behaviours in the current study. Those of non-white ethnicities were also found to have greater likelihood of carrying out hygiene behaviours in this study, as was found in general population surveys in other studies or previous pandemics [29, 61, 62].

Gender has been found as a hygiene determinant in many surveys, with students [39, 48], or general populations [61–63], but in the current study, the association was weaker than the other four determinants and did not reach significance, although was significant both in univariate association and in the initial model. It is possible that the influence of gender was manifest primarily through other determinants such as habit and time factors. These were found to have significant but modest negative correlations with gender in the full correlation matrix (*r*_*s*_ = − 0.2 and − 0.15 respectively, both *p* < 0.001). In addition, separate linear regressions for males and females, resulted in retention of time, habit and ethnicity for females, but only retention of habit for males, indicating a gender difference. The significant association of ethnicity in the linear regression but not in correlation analysis may be due to dependence on its effects through interactions with other variables included in the multivariate analysis. A key determinant for hygiene behaviour found in other surveys but not in this study, is perceived effectiveness of the behaviour [15, 42, 48, 63]. This may be due to differences in populations or due to the nature of the question in this survey, which was more as a knowledge question in response to the strong government health information messaging, than a question on gauging strength of belief in the effectiveness of the measure. Knowledge about the government’s message may not equate to strong enough belief to impact behaviour.

#### Determinants of social distancing behaviour

For social distancing, the trust construct was added to the standard motivational socio-cognitive constructs of attitude, social norms/support, and self-efficacy in the I-Change model, as a ‘predisposing’ factor. ‘Trust’ has been found to be a determinant of avoidance or social distancing behaviour in other studies of COVID-19 and previous pandemics [18, 42, 54]. The WHO longitudinal survey for COVID-19 captures many aspects of trust [21], but in this study, only indicative statements on trust in government policies affecting social distancing restrictions, and fairness of the policies were included. The bivariate analysis revealed significant correlations with social distancing behaviour of medium effect size for perceived self-efficacy, and small effect size for perceived advantages, social support and trust, all at *p* < 0.001. There were also small effect sizes but significant correlations for social distancing with gender and ethnicity as dichotomous variables, at *p* = 0.026. As with hand hygiene behaviours, knowledge of the effectiveness of the social distancing behaviour did not have a significant correlation with the behaviour itself.

Multi-variate analysis of determinants for social distancing behaviour confirmed self-efficacy as the greatest contributor to variance in behaviour, out of the I-Change motivational socio-cognitive constructs, followed by perceived advantage, and the predisposing factor ‘trust’, in this case trust in policy. Multiple linear regression is particularly sensitive to the presence and influence of the different variables and the order in which they are added to the model. Thus, the relative contributions of trust and advantages and also the significance, varied as the model was refined. However, in a final 4 variable model, self-efficacy, and trust made significant contributions at *p* < 0.001, with both advantages and gender at *p* < 0.05. The contribution of gender was confirmed when in separate linear regression analyses by gender, the female group retained self-efficacy and trust, with females assessed with higher scores in those factors more likely to practice the social distancing behaviours, while in males, no determinant was able to show a significant contribution. Self-efficacy [43, 54], trust [18, 42, 54], attitude or advantages [43, 64], and gender [54, 61, 64] have all been reported previously as determinants of avoidance or social distancing behaviour. In other studies, aspects of risk perception [41, 49, 59], perceived effectiveness of avoidance behaviours [18, 29, 42] and ethnicity [29, 62] have been shown as determinants for this behaviour, in contrast to this study. While for perceived effectiveness of measures, the difference may have been due to wording of question as a knowledge question, as described for hand hygiene behaviours, it is not known why risk perception and ethnicity were not significant in this study. Ethnicity contributions were significant before the addition of self-efficacy, advantages and social support to the model, so it is possible that ethnicity may act on social distancing behaviour through those determinants. Variations in populations, timing with respect to the pandemic, effectiveness of government communications, and the degree of impact of the pandemic may all affect the relative contribution of each potential determinant.

### Implications

The findings of this study have potential implications for both future public health interventions to address protective behaviours, as well as future research. At an overall behavioural level, lower rates of hand hygiene behaviour might imply that there is a greater need for interventions in this area than for social distancing. However, caution is required as this would also be influenced by the relative impact of the two behaviours on transmission of disease. The observation here and elsewhere that changing certain of the behaviour items such as face /mouth/nose touching may be particularly difficult [52], also means that understanding determinants of hand hygiene behaviours in order to better target interventions, gains particular importance.

In the I-Change Model, an awareness phase precedes the motivation phase, which can be directly influenced by distal predisposing factors. Then the motivation phase can lead to an action phase, which includes action planning, with a resulting change in behaviour [16]. Current approaches to health communications tend to focus on the awareness factors, knowledge and risk perception, but based on findings from this study, interventions for these protective behaviours during pandemics may need to focus on other predisposing factors to motivation, such as habit, time factors, and trust, as well as motivation factors e.g. self-efficacy. Therefore, early stages in the design of interventions, as recommended in Intervention Mapping [65], may need to include activities to better understand the potential of perceptions of these factors to impact the behaviour in the targeted population.

Identification of the significance of habit/automaticity and time factors for hand hygiene is particularly important, as addressing these determinants to enhance hand hygiene behaviour during pandemics may require different intervention methods to those addressed by health behaviour theories such as the Health Belief Model [66] and a different approach to health messages. Habits are typically developed at a young age but for changing adult behaviour, adaption of hand washing habit forming strategies devised for developing countries may need to be considered [67] as well as more general approaches to addressing habit-forming [65]. More innovative thinking may be needed to address the time factor determinant. Unlike in other studies, knowledge of the effectiveness of hand hygiene measures was not identified as a determinant. With a high profile public information campaign, and apparent high levels of knowledge of this effectiveness, it is possible that participants may have known the government message but not believed it strongly enough to impact their behaviour. Understanding this anomaly may also have an impact on health communication messaging. While in this survey practice of social distancing was high, it is possible that this may not be maintained as the pandemic progresses or in future waves [68], and so interventions may be required. As such there would be an increased need to address issues of trust and self-efficacy to improve this behaviour, even targeting by gender. While self-efficacy may be improved by health messaging and other techniques reviewed by Bartholemew Eldredge [65], trust in government policy may be a more complex construct to address.

Future research could benefit by increasing the sample size and expanding to the general UK population to surface additional potential associations and improve generalisability of the findings. In addition, longitudinal studies could be carried out, as both the frequency of the behaviours as well the relative importance of the determinants is likely to change over time with the changing face of the pandemic and government policies [69]. Already, changes to policy have included the use of face masks or coverings for the public, omitted intentionally from this study as they were against government policy at the time, but now should be included in such research. In addition, research on how to enhance perceptions of the factors found in this study, rather than just enhancing knowledge, is needed.

### Limitations

This study has a number of limitations. Initially it should be noted that this was a convenience sample of UK university students, as it involved participants who had chosen to be registered for the Prolific panel, and agreed to be surveyed at the time of the Prolific invitation. This was 97% of the those randomly selected. Use of such a convenience sample may limit ability to generalize findings, in addition to having inherent limitations from a smaller sample size and restricted sampling frame. It is also possible that participants randomly selected answers. However, no participants completed the survey in under 4 min, which would have been physically possible to do. In addition, checks were made for identical responses in multi-item Likert scales which would suggest non-attention, and attention-check questions were included [31]. There were 12 extreme outliers in the social distancing scale, which seemed to have discrepancies in related answers, and these were excluded from the relevant analysis dataset. These type of potential methodology issues were not considered an important source of bias.

Importantly, behaviours were all self-reported, which may result in a reporting bias. However, as the survey was anonymous, there would be less incentive to provide more socially acceptable answers. As this survey was cross-sectional in design, association is unable to imply causality.

A further limitation, in the interest of having a survey which could be completed in a reasonable timeframe, was that other potential determinants such as information/communication factors, social norms for hand hygiene, more comprehensive trust questions, worry, anxiety and transmission knowledge were omitted yet may also have been predictors of the behaviours. Further optimisation of wording of questions could also have been performed. For example, questions on behaviour gave options “Rarely/never” (or “Rarely/mostly”), “Mostly” or “Always” as the emphasis was on performing the behaviour sufficiently to be effective. Use of “Never/Sometimes” may have more accurately covered lower levels of behaviour.

Additionally, a number of scales have Cronbach’s alpha scores < 0.7 which would usually be assessed as low internal reliability, although Pallant [23] advises that for social sciences scores as low as 0. 5 may be found. Instead, for disadvantages and trust scales with α < 0.5, individual representative items were used, although these then become single item ordinal variables but have replaced continuous scale variables in a linear regression analysis. To compensate, these analyses have been reported both from the scales with the low alpha, as well as the individual items.

Variables assessed as scales did not show a normal distribution according to the Kolmogorov-Smirnov test, although visual inspection of many, especially the hand hygiene behaviour scale, suggested close to normal distribution. Although this led to the decision to use non-parametric tests and robust techniques for determining confidence intervals, it is possible that the distributions may not have been fully unimodal, with subgroups responding to the behaviours in different ways. This would have required other analytical techniques, such as the Bayesian Guassian mixture model, recently used by Wise [59].

Lastly, there can be different interpretations of the determinants in terms of their placement within the theoretical framework. Knowledge of the effectiveness of the behaviours in preventing the spread of infection could also be deemed a ‘perceived efficacy’ or a ‘perceived benefit’. However, the use of ‘Yes’, ‘No’, ‘Don’t know’ options from the WHO survey [21] rather than a Likert type agree-disagree scale, is likely more consistent with knowledge. Similarly, the time factors taken from Aunger [17] and assigned as ‘predisposing’ factors for the I-Change framework could be seen as ‘perceived cons’ and contributing to an attitude constructs. However, it was one of the highest of the determinants in Aunger’s model development for handwashing behaviour, was assessed separately and was greater than other individual ‘barriers’ which might otherwise be designated ‘perceived cons’.

## Conclusion

University students in the UK during the first wave of the COVID-19 pandemic had good levels of knowledge of the effectiveness of hand hygiene and social distancing in reducing transmission. However, while a high proportion of participants self-reported practicing social distancing behaviours, the practice of hand hygiene behaviours was more nuanced with certain activities such as washing hands before touching face, or after handling public equipment, more moderate.

The use of I-Change as the theoretical framework for this study enabled a wider range of determinants to be investigated, as well as typical pre-motivational and motivational factors such as knowledge, risk perception, attitude, social support and self-efficacy. The understanding from this study, that it is not knowledge, per se, but rather habit and time factors which are linked to hand hygiene behaviour, and self-efficacy and trust to social distancing behaviour, must surely have implications for those in authority, in devising appropriate interventions, including messages, to improve uptake of these behaviours. This is especially important in light of a likely second wave of infection, and when interest in compliance with social distancing may be on the wane [70]. The need to bring in the wearing of face coverings is yet another addition to the mix. Habit, time factors, and trust are complex determinants, without simplistic solutions, and so innovative thinking is required, together with a deeper understanding of these determinants in the UK university student population; such understanding may also result in implications for the wider population in the UK.

## Supplementary Information

**Additional file 1.** Hand hygiene and social distancing behaviour amongst UK university students during the COVID-19 pandemic Questionnaire.

**Additional file 2.** Supplementary Information. Supplementary Tables and Figure.

## Data Availability

The datasets generated and/or analysed during the current study are available in the figshare repository, 10.6084/m9.figshare.13172825.v1 .

## Abbreviations

BCa: Bias-corrected and accelerated
CI: Confidence interval
COVID-19: Coronavirus disease 19
I-Change: Integrated-Change
IQR: Inter-quartile range
NPI: Nonpharmaceutical intervention
*r*_*s*_: Spearman’s correlation coefficient
SARS-CoV-2: Severe acute respiratory syndrome coronavirus 2
SD: Standard deviation
UK: United Kingdom
WHO: World Health Organisation
