# Supplementary material for: Knowledge, socio-cognitive perceptions and the practice of hand hygiene and social distancing during the COVID-19 pandemic: a cross-sectional study of UK university students
Source: BMC Public Health. 2021 Mar 1;21:426. doi: 10.1186/s12889-021-10461-0 (PMC7919985; doi:10.1186/s12889-021-10461-0)
Supplement: Supplementary file 1 — Additional file 1. Hand hygiene and social distancing behaviour amongst UK university students during the COVID-19 pandemic Questionnaire. [file 12889_2021_10461_MOESM1_ESM.docx]

**
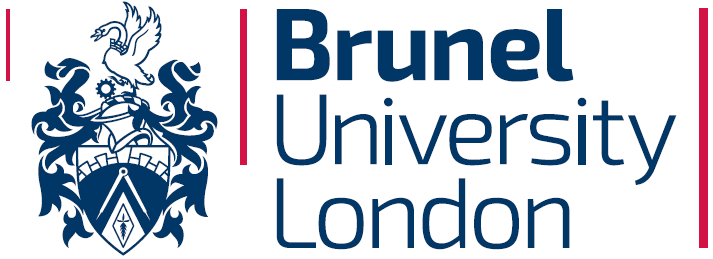
**Hand hygiene and social distancing behaviour amongst UK university students during the COVID-19 pandemic

**About you**

1. Are you currently a student at a UK University? ⭘ Yes ⭘ No

If you answered Yes, please continue

1. How old are you? I am _____ years old
2. What is your gender? ⭘ Male ⭘ Female ⭘ Other
3. Are you a UK or International student?

⭘ UK ⭘ International

1. Are you studying a Health/Life Science related course? ⭘ Yes ⭘ No
2. How would you describe your ethnicity?.

⭘ White

⭘ Black

⭘ Asian

⭘ Chinese

⭘ Mixed

⭘ Other

**Questions concerning the novel coronavirus, which causes COVID-19**

1. Are you or have you been infected with the novel coronavirus?

⭘ Yes, tested and the result was positive ⭘ Yes, suspected but not confirmed by a test
⭘ No, tested and the result was negative ⭘ No ⭘ Don’t know

1. Do you know people in your immediate social environment who are or have been infected with the novel coronavirus?

⭘ Yes, tested and the result was positive ⭘ Yes, suspected but not confirmed by a test
⭘ No, tested and the result was negative ⭘ No ⭘ Don’t know

1. Which groups are at risk of severe illness if they are infected with the novel coronavirus?

|  | Are at risk | Are not at risk | Don’t know |
| --- | --- | --- | --- |
| People aged 60 years or older | 🞏 | 🞏 | 🞏 |
| Small children aged 1-5 years | 🞏 | 🞏 | 🞏 |
| People who have asthma | 🞏 | 🞏 | 🞏 |
| People who have serious chronic heart disease | 🞏 | 🞏 | 🞏 |
| Pregnant women | 🞏 | 🞏 | 🞏 |
| People who have serious chronic diabetes | 🞏 | 🞏 | 🞏 |

1. Which of the following can be symptoms of the newly emerged coronavirus?

|  | Related | Not related | Don’t know |
| --- | --- | --- | --- |
| Fever | 🞏 | 🞏 | 🞏 |
| Runny or stuffy nose | 🞏 | 🞏 | 🞏 |
| Shortness of breath | 🞏 | 🞏 | 🞏 |
| Sore throat | 🞏 | 🞏 | 🞏 |
| Muscle or body aches | 🞏 | 🞏 | 🞏 |
| Cough | 🞏 | 🞏 | 🞏 |

1. Which answer is correct?

⭘ There is a vaccine to treat the novel coronavirus.

⭘ There is a drug for the novel coronavirus.

⭘ There is both a drug for the treatment and a vaccine for the novel coronavirus.

⭘ There is currently no drug treatment or vaccine for the novel coronavirus.

⭘ Don’t know

1. What is the maximum incubation period (i.e. the time from viral infection to developing symptoms of the illness) of the novel coronavirus?

⭘ Up to 3 days.

⭘ Up to 14 days.

⭘ Up to 7 days.

⭘ Don’t know

1. What do you consider to be your own probability of getting infected with the novel coronavirus?

Extremely unlikely 🞏 🞏 🞏 🞏 🞏 🞏 🞏 🞏 🞏 Extremely likely

1. How susceptible do you consider yourself to an infection with the novel coronavirus?

Not at all susceptible 🞏 🞏 🞏 🞏 🞏 🞏 🞏 🞏 🞏 Very susceptible

1. How severe would contracting the novel coronavirus be for you (how seriously ill do you think that you would be)?

Not severe 🞏 🞏 🞏 🞏 🞏 🞏 🞏 🞏 🞏 Very severe

1. For me avoiding an infection with the novel coronavirus in the current situation is…

Extremely difficult 🞏 🞏 🞏 🞏 🞏 🞏 🞏 🞏 🞏 Extremely easy

**Knowledge of hand hygiene practices such as handwashing**

1. Which of the following are effective hygiene or personal measures to prevent the spread and infection of the novel coronavirus?

|  | Yes | No | Don’t know |
| --- | --- | --- | --- |
| Hand washing with soap and water for at least 20 seconds | 🞏 | 🞏 | 🞏 |
| Avoiding touching your eyes, nose, and mouth with unwashed hands | 🞏 | 🞏 | 🞏 |
| Taking herbal supplements | 🞏 | 🞏 | 🞏 |
| Use of anti-bacterial gel to clean hands when soap and water is not available for washing hands | 🞏 | 🞏 | 🞏 |
| Covering your mouth with a tissue or sleeve when you cough or sneeze | 🞏 | 🞏 | 🞏 |
| Throwing away tissues immediately after coughing or sneezing and washing your hands | 🞏 | 🞏 | 🞏 |
| Putting your handkerchief in your pocket after coughing/sneezing | 🞏 | 🞏 | 🞏 |
| Washing hands thoroughly – palms, backs, between fingers, back and tips of fingers, thumbs | 🞏 | 🞏 | 🞏 |

**Your own hand hygiene practices related to the novel coronavirus**

1. I follow the guidance on handwashing and coughing/sneezing practice, to prevent spread of the novel coronavirus.

⭘ Yes ⭘ No

1. How do you wash your hands?

⭘ Water only ⭘ Soap and water (or anti-bacterial hand gel)

1. How long do you wash your hands for?

⭘ Less than 20 seconds ⭘ At least 20 seconds

1. How often do you practice the following hygiene activities?

|  | Rarely/never | Mostly | Always |
| --- | --- | --- | --- |
| Wash hands after using the toilet | 🞏 | 🞏 | 🞏 |
| Wash hands before eating or handling food | 🞏 | 🞏 | 🞏 |
| Wash hands after contact with animals or pets (leave blank if no contact) | 🞏 | 🞏 | 🞏 |
| Wash hands after handling money or public equipment (eg stair rails) | 🞏 | 🞏 | 🞏 |
| Wash hands after coughing or sneezing | 🞏 | 🞏 | 🞏 |
| Wash hands before touching face, eyes, nose, mouth | 🞏 | 🞏 | 🞏 |
| If sneeze or cough, then sneeze of cough into tissue or sleeve | 🞏 | 🞏 | 🞏 |
| Put tissues in the bin immediately after use | 🞏 | 🞏 | 🞏 |

1. Do you wash your hands more frequently or thoroughly than you did before the pandemic?

⭘ Yes ⭘ No ⭘ Don’t know

1. If relevant, what is your main reason for not washing hands?

⭘ Not used to it ⭘ Annoying ⭘ Not enough time

⭘ Other (please specify ) ______________

**Opinions and questions about other factors which may influence your hygiene practices**

1. What you may think of the benefits and disadvantages of hand hygiene practices and what factors influence some people.
   For each statement, please say whether you agree strongly, agree, are neutral, disagree or disagree strongly.

|  | Strongly agree | Agree | Neutral | Disagree | Strongly disagree |
| --- | --- | --- | --- | --- | --- |
| I believe that regular handwashing with soap and water will help prevent me from becoming infected with coronavirus | 🞏 | 🞏 | 🞏 | 🞏 | 🞏 |
| I like the feeling of washing my hands | 🞏 | 🞏 | 🞏 | 🞏 | 🞏 |
| I believe that regular handwashing with soap and water will help prevent vulnerable people from becoming infected with coronavirus | 🞏 | 🞏 | 🞏 | 🞏 | 🞏 |
| I believe that believe that covering my mouth and nose with a tissue or sleeve when I cough or sneeze will help prevent others from becoming infected with coronavirus | 🞏 | 🞏 | 🞏 | 🞏 | 🞏 |
| I think that regular handwashing with soap and water is too much effort | 🞏 | 🞏 | 🞏 | 🞏 | 🞏 |
| I think that washing my hands can hurt them | 🞏 | 🞏 | 🞏 | 🞏 | 🞏 |
| I think that covering my mouth and nose with a tissue or sleeve when I cough or sneeze, is too much effort | 🞏 | 🞏 | 🞏 | 🞏 | 🞏 |
| Even if I am busy, I manage to wash my hands with soap after the toilet | 🞏 | 🞏 | 🞏 | 🞏 | 🞏 |
| It takes too much time to wash my hands with soap each time I prepare food | 🞏 | 🞏 | 🞏 | 🞏 | 🞏 |
| Hand-washing with soap and water for 20 seconds is quick and very easy to do | 🞏 | 🞏 | 🞏 | 🞏 | 🞏 |
| I sometimes start washing my hands with soap without even realizing I'm doing it. | 🞏 | 🞏 | 🞏 | 🞏 | 🞏 |
| I feel strange when I don’t wash my hands with soap after the toilet | 🞏 | 🞏 | 🞏 | 🞏 | 🞏 |
| Washing my hands with soap before I eat a meal is something I do automatically | 🞏 | 🞏 | 🞏 | 🞏 | 🞏 |
| If I feel I am going to cough or sneeze, I have a tissue or my sleeve ready to cover my mouth and nose without even realizing I’m doing it | 🞏 | 🞏 | 🞏 | 🞏 | 🞏 |
| I am confident that I can practise handwashing correctly when at home | 🞏 | 🞏 | 🞏 | 🞏 | 🞏 |
| I am confident that I can practise handwashing correctly when away from home | 🞏 | 🞏 | 🞏 | 🞏 | 🞏 |
| I am confident that I can practise good cough etiquette | 🞏 | 🞏 | 🞏 | 🞏 | 🞏 |

**Knowledge of social distancing practices**

1. Which of the following are effective social distancing measures to prevent the spread and infection of the novel coronavirus?

|  | Yes | No | Don’t know |
| --- | --- | --- | --- |
| Staying at home when you are sick or have a cold | 🞏 | 🞏 | 🞏 |
| Meeting with friends and family outside the home | 🞏 | 🞏 | 🞏 |
| Avoiding close contact with someone who is infected | 🞏 | 🞏 | 🞏 |
| Avoiding places where people gather | 🞏 | 🞏 | 🞏 |
| Going to get essential groceries at the shops as little as possible | 🞏 | 🞏 | 🞏 |
| Keeping at least 2 metres from other people when you are outside the home | 🞏 | 🞏 | 🞏 |
| Visiting the elderly or those who are sick | 🞏 | 🞏 | 🞏 |
| Limiting exercise outside your home to once a day and alone or with members of your household | 🞏 | 🞏 | 🞏 |
| Staying at home for 14 days after someone in your home starts having high temperature or new continuous cough | 🞏 | 🞏 | 🞏 |
| Staying at home for 7 days or till symptoms stop if you develop COVID-19 symptoms of high temperature or new continuous cough | 🞏 | 🞏 | 🞏 |

**Your own social distancing practices related to the novel coronavirus.**

1. I follow the recommendations from the authorities on practicing social distancing to prevent spread of the novel coronavirus.

⭘ Yes ⭘ No

1. How well do you comply with the following social distancing guidance?

|  | Rarely/mostly | Mostly | Always |
| --- | --- | --- | --- |
| Staying at home when sick or have a cold (or intending to stay if situation occurs) | 🞏 | 🞏 | 🞏 |
| Isolating at home if have high temperature or new, continuous cough until symptoms stop or at least 7 days (or intending to isolate if situation occurs) | 🞏 | 🞏 | 🞏 |
| Isolating at home for 14 days if someone in household develops high temperature or new, continuous cough (or intending to isolate if situation occurs) | 🞏 | 🞏 | 🞏 |
| Shopping only for basic necessities, as infrequently as possible | 🞏 | 🞏 | 🞏 |
| Restricting to one form of outside exercise a day alone or with members of your household (or as advised by authorities) | 🞏 | 🞏 | 🞏 |
| Not meeting in groups | 🞏 | 🞏 | 🞏 |
| Staying at home except for essential shopping, exercise, medical need or work where you cannot work from home | 🞏 | 🞏 | 🞏 |
| If outside the house (i.e. for allowed reasons), staying at least 2 m away from others not in household | 🞏 | 🞏 | 🞏 |

**Opinions and questions about other factors which may influence how you practice social distancing**

1. What you may think of the benefits and disadvantages of social distancing and what factors influence some people.
   For each statement, please say whether you agree strongly, agree, are neutral, disagree or disagree strongly.

|  | Strongly agree | Agree | Neutral | Disagree | Strongly disagree |
| --- | --- | --- | --- | --- | --- |
| I believe social distancing will help protect me from getting the novel coronavirus | 🞏 | 🞏 | 🞏 | 🞏 | 🞏 |
| I believe social distancing will help protect the vulnerable from getting coronavirus | 🞏 | 🞏 | 🞏 | 🞏 | 🞏 |
| I believe that social distancing will protect the NHS from having too many cases of COVID-19 in hospital at once | 🞏 | 🞏 | 🞏 | 🞏 | 🞏 |
| I think I miss meeting up with family and friends | 🞏 | 🞏 | 🞏 | 🞏 | 🞏 |
| I think I (or someone close to me) will lose my/their job (or have lost my job and will not be able to find other work) | 🞏 | 🞏 | 🞏 | 🞏 | 🞏 |
| I think I will get too bored during the time of social distancing | 🞏 | 🞏 | 🞏 | 🞏 | 🞏 |
| My family and friends avoid crowded areas | 🞏 | 🞏 | 🞏 | 🞏 | 🞏 |
| My family and friends avoid social contacts | 🞏 | 🞏 | 🞏 | 🞏 | 🞏 |
| My friends encourage me to meet with them, against government guidelines | 🞏 | 🞏 | 🞏 | 🞏 | 🞏 |
| I think the authorities should restrict personal liberty rights to combat the novel coronavirus | 🞏 | 🞏 | 🞏 | 🞏 | 🞏 |
| I think the decisions that have been made to reduce the spread of the novel coronavirus are fair | 🞏 | 🞏 | 🞏 | 🞏 | 🞏 |
| I think the authorities should relax the restrictions even if there are still many new cases of COVID-19 emerging | 🞏 | 🞏 | 🞏 | 🞏 | 🞏 |
| I am confident that I can practise social distancing from family and friends who do not live in my household | 🞏 | 🞏 | 🞏 | 🞏 | 🞏 |
| I am confident that I can practise social distancing when I am outside for essential activities | 🞏 | 🞏 | 🞏 | 🞏 | 🞏 |
| I am confident that I can practise social distancing when I am outside for exercise | 🞏 | 🞏 | 🞏 | 🞏 | 🞏 |
