# Supplementary material for: Knowledge, socio-cognitive perceptions and the practice of hand hygiene and social distancing during the COVID-19 pandemic: a cross-sectional study of UK university students
Source: BMC Public Health. 2021 Mar 1;21:426. doi: 10.1186/s12889-021-10461-0 (PMC7919985; doi:10.1186/s12889-021-10461-0)
Supplement: Supplementary file 2 — Additional file 2. Supplementary Information. Supplementary Tables and Figure. [file 12889_2021_10461_MOESM2_ESM.docx]

# **Supplementary Information**

Knowledge, socio-cognitive perceptions and the practice of hand hygiene and social distancing during the COVID-19 pandemic: A cross-sectional study of UK university students

Christine Barrett, Kei Long Cheung

**Table S1: Frequency of hand hygiene behaviour activities**

| **How often do you practise the following activities?** | ***N*** | **Rarely/never**  ***n* (%)** | **Mostly**  ***n* (%)** | **Always**  ***n* (%)** |
| --- | --- | --- | --- | --- |
| Wash hands after using the toilet | 293 | 4 (1.4) | 48 (16.4) | 241 (82.3) |
| Wash hands before eating or handling food | 293 | 25(8.5) | 146 (49.8) | 122 (41.6) |
| Wash hands after contact with animals or pets (blank if no contact) | 229 | 71 (24.2) | 66 (22.5) | 92 (31.4) |
| Wash hands after handling money or public equipment | 293 | 62 (21.2) | 98 (33.4) | 133 (45.4) |
| Wash hands after coughing or sneezing | 292 | 59 (20.1) | 125 (42.7) | 108 (36.9) |
| Wash hands before touching face, eyes, nose, mouth | 293 | 129 (44.0) | 109 (37.2) | 55 (18.8) |
| If sneeze or cough, then sneeze of cough into tissue or sleeve | 293 | 33 (11.3) | 94 (32.1) | 166 (56.7) |
| Put tissues in the bin immediately after use | 293 | 18 (6.1) | 101 (34.5) | 174 (59.4) |
| Hand hygiene behaviour score - mean (SD)/ median (IQR) ^a^ | 293 | 1.78 (± 0.69)/ 1.75 (1.0) | | |

Abbreviations: SD, standard deviation; IQR, interquartile range

^a^ Hand hygiene behaviour score was normally distributed by visual inspection of histogram but was significantly different from a normal distribution by the Kolmogorov-Smirnov test (*p* = 0.035). Both mean and median measure of central tendency are shown.

**Table S2: Frequency of social distancing behaviour activities**

| **How often do you practise the following activities?** | ***N*** | **Rarely/mostly**  ***n* (%)** | **Mostly**  ***n* (%)** | **Always**  ***n* (%)** |
| --- | --- | --- | --- | --- |
| Staying at home when sick or have a cold | 279 | 2 (0.7) | 26 (9.3) | 251 (90.0) |
| Isolating at home if have high temperature or new, continuous cough until symptoms stop or at least 7 days | 280 | 3 (1.1) | 13 (4.6) | 264 (94.3) |
| Isolating at home for 14 days if someone in household develops high temperature or new, continuous cough | 280 | 3 (1.1) | 23 (7.8) | 255 (91.1) |
| Shopping only for basic necessities, as infrequently as possible | 280 | 4 (1.4) | 80 (28.6) | 196 (70.0) |
| Restricting to one form of outside exercise a day alone or with members of your household | 281 | 7 (2.5) | 62 (22.1) | 212 (75.4) |
| Not meeting in groups | 281 | 3 (1.1) | 12 (4.3) | 266 (94.7) |
| Staying at home except for essential shopping, exercise, medical need or work where you cannot work from home | 280 | 3 (1.1) | 34 (12.1) | 243 (86.8) |
| If outside the house, staying at least 2 m away from others not in household | 281 | 4 (1.4) | 61 (21.7) | 216 (76.9) |
| Social distancing behaviour score median (IQR) ^a^ | 281 | 2.75 (0.5) | | |

Abbreviations: SD, standard deviation; IQR, interquartile range

^a^ Social distancing behaviour score was not normally distributed by visual inspection of histogram and was significantly different from a normal distribution by the Kolmogorov-Smirnov test (*p* < 0.001), indicating median should be used. Mean (SD): 2.69 (0.44).

**Table S3: Risk perception: probability, susceptibility and severity**

| **Risk Perception – *n* (%)** | **1** | **2** | | **3** | **4** | **5** | **6** | **7** | **8** | **9** |  |
| --- | --- | --- | --- | --- | --- | --- | --- | --- | --- | --- | --- |
|  | Extremely unlikely Extremely likely | | | | | | | | | |  |
| Probability | 12  (4.1) | 47  (16.0) | | 53  (18.1) | 42  (14.3) | 44  (15.0) | 48  (16.4) | 31  (10.6) | 10  (3.4) | 6  (2.0) |  |
|  | Not at all susceptible Very susceptible | | | | | | | | | |  |
| Susceptibility | 10  (3.4) | 47  (16.0) | | 56  (19.1) | 44  (15.0) | 52  (17.7) | 31  (10.6) | 33  (11.3) | 14  (4.8) | 6  (2.0) |  |
|  | Not severe Very severe | | | | | | | | | |  |
| Severity | 25  (8.5) | 54  (18.4) | | 59  (20.1) | 39  (13.3) | 30  (10.2) | 35  (11.9) | 29  (9.9) | 18  (6.1) | 4  (1.4) |  |
| Overall risk perception score – mean (SD)/median (IQR) ^a^ | | | 4.31 (±1.55)/ 4.33 (2.0) | | | | | | | | |

^a^ Risk perception score was approximately normally distributed by visual inspection of histogram but was significantly different from a normal distribution by the Kolmogorov-Smirnov test (*p* = 0.001). Both mean and median measure of central tendency are shown.

**Figure S1: Participant responses for knowledge of COVID-19 risk groups and symptoms**

**
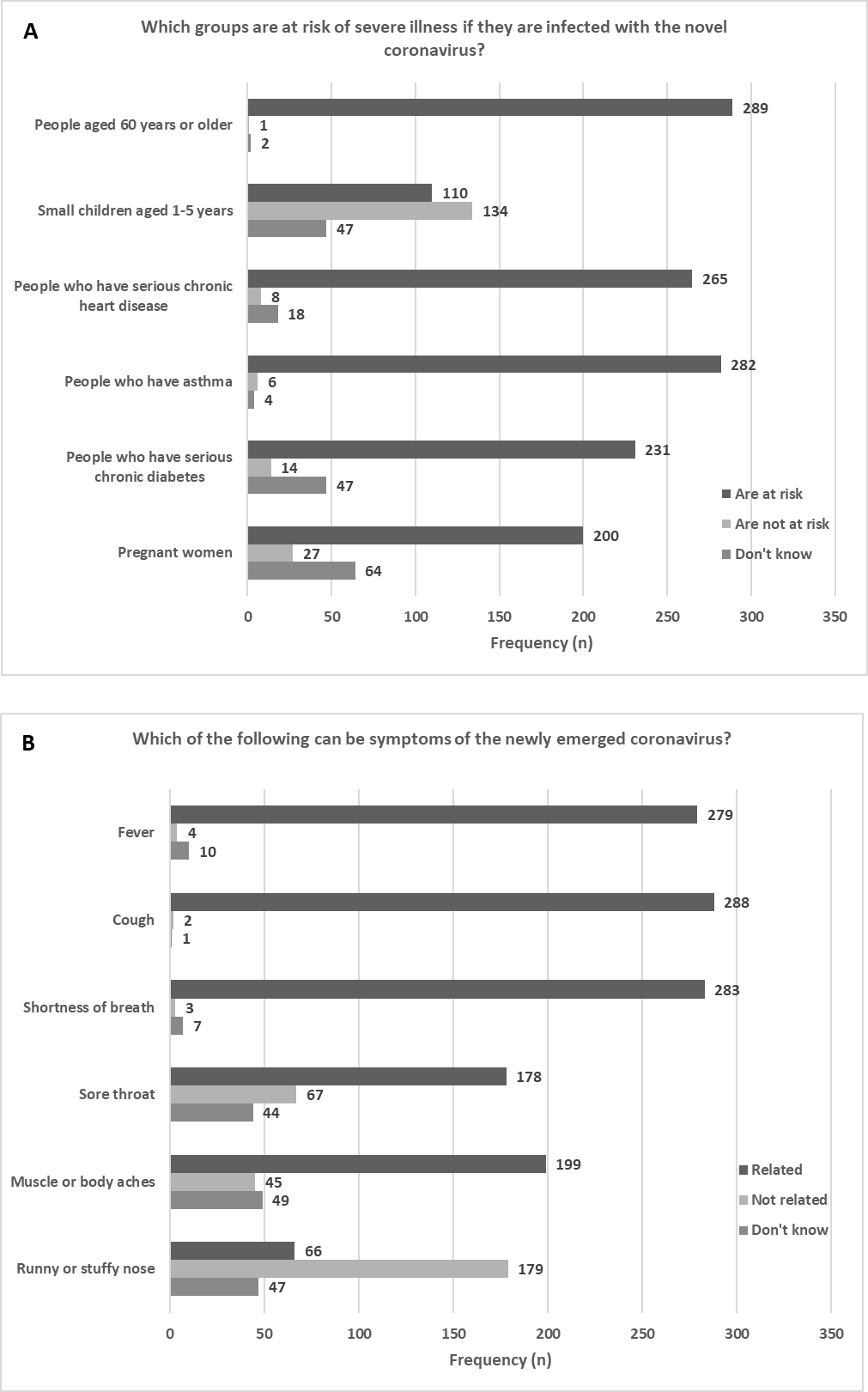
**

**Table S4: Attitudes, self-efficacy, time factors and habit in hand hygiene behaviour**

| **Domain and belief** | ***N*** | **Strongly agree**  ***n*(%)** | **Agree**  ***n* (%)** | **Neutral**  ***n* (%)** | **Disagree**  ***n* (%)** | **Strongly disagree**  ***n* (%)** |
| --- | --- | --- | --- | --- | --- | --- |
| **Attitude (Advantages)** | | | | | | |
| I believe that regular handwashing with soap and water will help prevent me from becoming infected with coronavirus | 293 | 134  (45.7) | 135  (46.1) | 17  (5.8) | 7  (2.4) | 0  (0) |
| I like the feeling of washing my hands | 293 | 60  (20.5) | 111 (37.9) | 91 (31.1) | 25 (8.5) | 6  (2.0) |
| I believe that regular handwashing with soap and water will help prevent vulnerable people from becoming infected with coronavirus | 293 | 114  (38.9) | 139  (47.4) | 26  (8.9) | 12  (4.1) | 2  (0.7) |
| I believe that covering my mouth and nose with a tissue or sleeve when I cough or sneeze will help prevent others from becoming infected with coronavirus | 293 | 161  (54.9) | 107  (36.5) | 19  (6.5) | 6  (2.0) | 0  (0) |
| **(Attitude) Disadvantages** | | | | | | |
| I think that regular handwashing with soap and water is too much effort | 293 | 9  (3.1) | 20  (6.8) | 27  (9.2) | 104  (35.5) | 133  (45.4) |
| I think that washing my hands can hurt them | 293 | 16  (5.5) | 63  (21.5) | 50  (17.1) | 79  (27.0) | 85  (29.0) |
| I think that covering my mouth and nose with a tissue or sleeve when I cough or sneeze, is too much effort | 292 | 3  (1.0) | 11  (3.8) | 14  (4.8) | 70  (23.9) | 194  (66.2) |
| **Time factor** | | | | | | |
| Even if I am busy, I manage to wash my hands with soap after the toilet | 293 | 194  (66.2) | 69  (23.5) | 21  (7.2) | 8  (2.7) | 1  (0.3) |
| It takes too much time to wash my hands with soap each time I prepare food | 293 | 6  (2.0) | 27  (9.2) | 48  (16.4) | 96  (32.8) | 116  (39.6) |
| Hand-washing with soap and water for 20 seconds is quick and very easy to do | 293 | 138  (47.1) | 103  (35.2) | 29  (9.9) | 20  (6.8) | 3  (1.0) |
| **Habit/automaticity** | | | | | | |
| I sometimes start washing my hands without even realizing I’m doing it | 291 | 107  (36.5) | 83  (28.3) | 32  (10.9) | 44  (15.0) | 25  (8.5) |
| I feel strange when I don’t wash my hands with soap after the toilet | 292 | 165  (56.3) | 61  (20.8) | 35  (11.9) | 25  (8.5) | 6  (2.0) |
| Washing my hands with soap before I eat a meal is something I do automatically | 293 | 67  (22.9) | 71  (24.2) | 55  (18.8) | 73  (24.9) | 27  (9.2) |
| If I feel I am going to cough or sneeze, I have a tissue or my sleeve ready to cover my mouth and nose without even realizing I’m doing it | 293 | 98  (33.4) | 101  (34.5) | 39  (13.3) | 45  (15.4) | 10  (3.4) |
| **Self-Efficacy – Hand hygiene** | | | | | | |
| I am confident that I can practise handwashing correctly when at home | 293 | 195  (66.6) | 78  (26.6) | 16  (5.5) | 3  (1.0) | 1  (0.3) |
| I am confident that I can practise handwashing correctly when away from home | 293 | 157  (53.6) | 88  (30.0) | 22  (7.5) | 25  (8.5) | 1  (0.3) |
| I am confident that I can practise good cough etiquette | 293 | 184  (62.8) | 87  (29.7) | 19  (6.5) | 3  (1.0) | 0 |

**Table S5: Attitudes, social support, self-efficacy, and trust in social distancing behaviour**

| **Domain and belief** | ***N*** | **Strongly agree**  ***n* (%)** | **Agree**  ***n* (%)** | **Neutral**  ***n* (%)** | **Disagree**  ***n* (%)** | **Strongly disagree**  ***n* (%)** |
| --- | --- | --- | --- | --- | --- | --- |
| **Advantages** | | | | | | |
| I believe social distancing will help protect me from getting the novel coronavirus | 293 | 174  (59.4) | 101  (34.8) | 12  (4.1) | 4  (1.4) | 1  (0.3) |
| I believe social distancing will help protect the vulnerable from getting coronavirus | 293 | 201  (68.6) | 79  (27.0) | 10  (3.4) | 2  (0.7) | 1  (0.3) |
| I believe that social distancing will protect the NHS from having too many cases of COVID-19 in hospital at once | 292 | 198  (67.8) | 83  (28.4) | 6  (2.1) | 3  (1.0) | 2  (0.7) |
| **Disadvantages** | | | | | | |
| I think I miss meeting up with family and friends | 291 | 173  (59.5) | 69  (23.7) | 28  (9.6) | 14  (4.8) | 7  (2.4) |
| I think I (or someone close to me) will lose my job | 293 | 72  (24.6) | 101  (34.5) | 59  (20.1) | 47  (16.0) | 14  (4.8) |
| I think I will get too bored during the time of social distancing | 292 | 64  (21.9) | 82  (28.1) | 52  (17.8) | 68  (23.3) | 26  (8.9) |
| **Social Support** | | | | | | |
| My family and friends avoid crowded areas | 291 | 165  (56.7) | 105  (35.8) | 15  (5.2) | 5  (1.7) | 1  (0.3) |
| My family and friends avoid social contacts | 290 | 154  (53.1) | 106  (36.6) | 21  (7.2) | 7  (2.4) | 2  (0.7) |
| My friends encourage me to meet with them, against government guidelines | 293 | 12  (4.1) | 24  (8.2) | 11  (3.8) | 78  (26.6) | 168  (57.3) |
| **Trust** | | | | | | |
| I think the authorities should restrict personal liberty rights to combat the novel coronavirus | 292 | 47  (16.1) | 85  (29.1) | 94  (32.3) | 47  (16.1) | 19  (6.5) |
| I think the decisions that have been made to reduce the spread of the novel coronavirus are fair | 293 | 118  (40.3) | 118  (40.3) | 30  (10.2) | 20  (6.8) | 7  (2.4) |
| I think the authorities should relax the restrictions even if there are still many new cases of COVID-19 emerging | 293 | 8  (2.7) | 26  (8.9) | 26  (8.9) | 68  (23.2) | 165  (56.3) |
| **Self-efficacy** | | | | | | |
| I am confident that I can practise social distancing from family and friends who do not live in my household | 293 | 177  (60.4) | 90  (30.7) | 17  (5.8) | 6  (2.0) | 3  (1.0) |
| I am confident that I can practise social distancing when I am outside for essential activities | 292 | 139  (47.6) | 106  (36.3) | 27  (9.2) | 17  (5.8) | 3  (1.0) |
| I am confident that I can practise social distancing when I am outside for exercise | 293 | 166  (56.7) | 100  (34.1) | 16  (5.5) | 10  (3.4) | 1  (0.3) |

**Table S6: Correlation Matrix for general and hand hygiene related variables**

|  | (1) | (2) | (3) | (4) | (5) | (6) | (7) | (8) | (9) | (10) | (11) | (12) | (13) | (14) | (15) | (16) | (17) | (18) |
| --- | --- | --- | --- | --- | --- | --- | --- | --- | --- | --- | --- | --- | --- | --- | --- | --- | --- | --- |
| Age (1) | 1.00 |  |  |  |  |  |  |  |  |  |  |  |  |  |  |  |  |  |
| Gender (Male/Female) (2) | -0.09 | 1.00 |  |  |  |  |  |  |  |  |  |  |  |  |  |  |  |  |
| Ethnicity (White/Minorities) (3) | 0.05 | -0.06 | 1.00 |  |  |  |  |  |  |  |  |  |  |  |  |  |  |  |
| Student Status (UK/International) (4) | 0.11 | 0.03 | **-.14^*^** | 1.00 |  |  |  |  |  |  |  |  |  |  |  |  |  |  |
| Health Course (5) | 0.04 | -0.11 | **-.12^*^** | -0.07 | 1.00 |  |  |  |  |  |  |  |  |  |  |  |  |  |
| Infection exposure (6) | 0.06 | 0.02 | 0.00 | -0.07 | -.02 | 1.00 |  |  |  |  |  |  |  |  |  |  |  |  |
| Disease knowledge (7) | 0.08 | -0.09 | 0.06 | -0.03 | 0.00 | -0.01 | 1.00 |  |  |  |  |  |  |  |  |  |  |  |
| Risk perception (8) | **.14^*^** | -0.11 | 0.08 | -0.08 | 0.05 | **.24^**^** | 0.03 | 1.00 |  |  |  |  |  |  |  |  |  |  |
| Self-efficacy- infection avoidance (9) | **-.14^*^** | 0.04 | -0.03 | 0.08 | -.01 | **-.19^**^** | -0.02 | **-.42^**^** | 1.00 |  |  |  |  |  |  |  |  |  |
| Hand hygiene knowledge (10) | 0.02 | -0.01 | **.17^**^** | -0.10 | -.08 | -0.03 | -0.03 | 0.02 | 0.01 | 1.00 |  |  |  |  |  |  |  |  |
| Hand Hygiene Compliance (11) | 0.03 | -0.10 | 0.01 | 0.03 | 0.00 | -0.01 | 0.07 | **.15^**^** | -0.06 | -.01 | 1.00 |  |  |  |  |  |  |  |
| Hand hygiene behaviour (12) | 0.01 | **-.23^**^** | -0.08 | -0.07 | 0.04 | 0.11 | 0.10 | **.20^**^** | -0.08 | -.07 | **.26^**^** | 1.00 |  |  |  |  |  |  |
| Attitude hand hygiene (13) | -0.03 | -0.10 | -0.06 | 0.01 | -.01 | -0.01 | 0.06 | -0.05 | **0.12^*^** | 0.10 | **.13^*^** | **.26^**^** | 1.00 |  |  |  |  |  |
| Advantages hand hygiene (14) | -0.05 | -0.09 | -0.07 | -0.06 | 0.01 | -0.04 | 0.01 | 0.03 | 0.04 | 0.06 | **.12^*^** | **.29^**^** | **.69^**^** | 1.00 |  |  |  |  |
| Disadvantages (reversed) (15) | -0.01 | -0.06 | -0.04 | 0.04 | -.02 | 0.03 | 0.06 | -0.07 | **0.13^*^** | 0.03 | 0.11 | **.19^**^** | **.84^**^** | **.23^**^** | 1.00 |  |  |  |
| Time factors hand hygiene (16) | -0.01 | **-.15^**^** | 0.05 | **-.15^*^** | 0.03 | 0.06 | **0.12^*^** | 0.01 | 0.03 | 0.02 | **.30^**^** | **.49^**^** | **.51^**^** | **.34^**^** | **.45^**^** | 1.00 |  |  |
| Habit hand hygiene score (17) | -0.01 | **-.20^**^** | -0.04 | -0.09 | 0.00 | 0.06 | 0.08 | 0.10 | 0.00 | -.04 | **.22^**^** | **.58^**^** | **.37^**^** | **.35^**^** | **.28^**^** | **.60^**^** | 1.00 |  |
| Self-efficacy hand hygiene score (18) | 0.00 | **-.17^**^** | 0.00 | **-.13^*^** | 0.11 | 0.10 | 0.06 | 0.07 | -0.06 | 0.00 | **.23^**^** | **.38^**^** | **.47^**^** | **.38^**^** | **.36^**^** | **.54^**^** | **.47^**^** | 1.00 |

Bold: * *p* <0.05 (significant, 2-tailed); ** *p* <0.01 (significant, 2-tailed)

**Table S7: Correlation Matrix for general and social distancing related variables**

|  | (1) | (2) | (3) | (4) | (5) | (6) | (7) | (8) | (9) | (10) | (11) | (12) | (13) | (14) | (15) | (16) | (17) |
| --- | --- | --- | --- | --- | --- | --- | --- | --- | --- | --- | --- | --- | --- | --- | --- | --- | --- |
| Age (1) | 1.00 |  |  |  |  |  |  |  |  |  |  |  |  |  |  |  |  |
| Gender (Male/Female) (2) | -0.09 | 1.00 |  |  |  |  |  |  |  |  |  |  |  |  |  |  |  |
| Ethnicity (White/Minorities) (3) | 0.07 | -0.07 | 1.00 |  |  |  |  |  |  |  |  |  |  |  |  |  |  |
| Student Status (UK/International) (4) | 0.11 | 0.04 | **-.14^*^** | 1.00 |  |  |  |  |  |  |  |  |  |  |  |  |  |
| Health Course (5) | 0.03 | -0.11 | **-.13*** | -0.07 | 1.00 |  |  |  |  |  |  |  |  |  |  |  |  |
| Infection exposure (6) | 0.06 | 0.03 | 0.02 | -0.08 | -.03 | 1.00 |  |  |  |  |  |  |  |  |  |  |  |
| Disease knowledge (7) | **0.12^*^** | -0.09 | 0.05 | -0.03 | 0.02 | -0.01 | 1.00 |  |  |  |  |  |  |  |  |  |  |
| Risk perception (8) | **0.12^*^** | -0.11 | 0.08 | -0.08 | 0.05 | **0.26^**^** | 0.06 | 1.00 |  |  |  |  |  |  |  |  |  |
| Self-efficacy- infection avoidance (9) | **-.14^*^** | 0.04 | -0.04 | 0.07 | -.01 | **-.20^**^** | -0.03 | **-.42^**^** | 1.00 |  |  |  |  |  |  |  |  |
| Social distancing knowledge (10) | 0.05 | **-.15^*^** | 0.12 | -0.05 | -.06 | -0.04 | 0.09 | 0.05 | -0.02 | 1.00 |  |  |  |  |  |  |  |
| Social Distancing compliance (11) | -0.01 | -0.02 | -0.11 | -0.03 | 0.02 | 0.01 | -0.07 | 0.09 | 0.02 | -0.11 | 1.00 |  |  |  |  |  |  |
| Social distancing behaviour (12) | 0.03 | **-.13^*^** | -.124 | -0.03 | 0.02 | 0.01 | 0.06 | 0.07 | **0.12^*^** | 0.11 | **0.27^**^** | 1.00 |  |  |  |  |  |
| Advantages social distancing (13) | -0.01 | -0.02 | -0.10 | 0.07 | -.02 | -0.06 | 0.06 | 0.03 | **0.15^*^** | **0.17^**^** | **0.17^**^** | **0.26^**^** | 1.00 |  |  |  |  |
| Disadvantages (reversed) (14) | 0.11 | **0.15^*^** | -0.01 | **0.16^**^** | 0.01 | **-.16^**^** | -.13^*^ | -0.02 | -0.02 | 0.05 | 0.06 | 0.02 | -0.01 | 1.00 |  |  |  |
| Social support social distancing (15) | -0.06 | **-.13^*^** | 0.05 | -0.05 | 0.04 | 0.01 | .120^*^ | 0.03 | 0.09 | **0.17^**^** | 0.10 | **0.28^**^** | **0.28^**^** | 0.01 | 1.00 |  |  |
| Trust social distancing score (16) | 0.05 | -0.09 | **0.14^*^** | -0.09 | 0.05 | 0.06 | 0.09 | **0.12^*^** | 0.09 | **0.21^**^** | **0.18^**^** | **0.28^**^** | **0.29^**^** | 0.04 | **0.27^**^** | 1.00 |  |
| Self-efficacy social distancing (17) | -0.02 | -0.02 | -0.09 | -0.09 | 0.08 | 0.03 | -0.03 | 0.04 | 0.06 | **0.14^*^** | **0.18^**^** | **0.43^**^** | **0.37^**^** | -.02 | **0.39^**^** | **0.26^**^** | 1.00 |

Bold: * *p* <0.05 (significant, 2-tailed); ** *p* <0.01 (significant, 2-tailed)
